# Assessment of a novel patient reported outcome measure for visual snow syndrome: the Colorado visual snow survey 2.0

**DOI:** 10.3389/fneur.2025.1664310

**Published:** 2025-09-29

**Authors:** Samuel M. Maione, Victoria S. Pelak, Peter Gerhardstein

**Affiliations:** ^1^Psychological and Brain Sciences, Johns Hopkins University, Baltimore, MD, United States; ^2^Department of Psychology, Binghamton University, Binghamton, NY, United States; ^3^Departments of Neurology and Ophthalmology, University of Colorado School of Medicine, Aurora, CO, United States

**Keywords:** visual snow syndrome, patient reported outcome measure, epidemiology, persistent positive visual phenomena, Colorado visual snow survey

## Abstract

**Introduction:**

Visual snow syndrome (VSS) is a condition in which people experience a continuous overlay of small dots atop their entire visual field. As a newly recognized condition, there is a gap in patient reported outcome measures (PROMs) that target VSS symptom impact.

**Methods:**

We sought to assess the Colorado Visual Snow Survey 2.0 (CVSS) as a possible PROM for VSS using a convenience sample of undergraduate students and people with VSS recruited through the Visual Snow Initiative (N = 144).

**Results:**

We found the CVSS (1) strongly differentiated people with VSS from healthy controls, (2) demonstrated high internal consistency, and (3) aside from visual static, the degree of night vision impairment, blue field entoptic phenomenon, and afterimages, and tinnitus (in that order) best predicted group membership. We also find evidence to suggest people with VSS may be more sensitive to entoptic phenomenon and depersonalization/derealization than control participants.

**Conclusion:**

Overall, CVSS is a promising PROM that warrants further validation.

## 1 Introduction

Visual snow syndrome (VSS) is a relatively newly recognized neurological syndrome characterized by the continuous perception of an overlay of small dots throughout the entire visual field, similar to the static of an analog television not tuned to a channel (1). Typically, the symptoms are binocular and black and white, although some report monocular or multicolored perceptions. Additional symptoms include tinnitus, palinopsia (to include herein both visual afterimages and visual trails of moving objects), blue-field entoptic phenomena, floaters, flashes, nyctalopia, and photophobia. Associated symptoms are not uniformly present in every patient with VSS (2). VSS is strongly associated with a medical history of migraine headaches and migraine aura (19). The International Classification of Headache Disorders (ICHD-3) criteria for VSS are shown in Table 1 (20). The degree of dysfunction and disability experienced from each symptom of VSS is variable, resulting in challenges for the design of outcome measures for treatment trials.

**Table 1:** Summary of ICHD-3 criteria for VSS (20).

Dynamic, continuous, tiny dots across the entire visual field, persisting for >3 months Additional visual symptoms of at least two of the following four types: Palinopsia Enhanced entoptic phenomena Photophobia Impaired night vision (nyctalopia) Symptoms are not consistent with typical migraine visual aura Symptoms are not better accounted for by another disorder

The ICHD-3 criteria are pertinent for diagnostic purposes, while measures of symptom intensity and disability have not been adequately investigated. There remains a gap in patient-reported outcome measures (PROMs) designed to capture the severity of each VSS symptom and the impact of each symptom on function. The Colorado Visual Snow Survey (CVSS) was designed to fill this gap and was first published in 2021 [first version (3)] as a novel PROM for VSS-associated symptoms and disability over time. The initial version of the CVSS was recently redesigned (version 2.0) to improve the intra-rater reliability of the first version (non-published data), and was used for the present study.

Part 1 of the CVSS is a self-report of the presence or absence of core VSS symptoms and other symptoms commonly comorbid with VSS. Then, four metrics for assessment are additionally inquired upon if the symptom is present. Symptoms include visual static, afterimages, trails, blue field entoptic phenomenon, floaters, night vision impairment, tinnitus, depersonalization and derealization, anxiety, and sadness (or loss of interest in activities usually enjoyed). These symptoms were chosen from experiences with patients in clinical practice, and because these symptoms demonstrate abnormally high prevalence rates in people with VSS (2, 4). Symptoms are reported as either present or absent over the past 1 month. For each symptom that is present, four questions are used to create four symptom metrics as follows: one metric for symptom *intensity* (e.g., “In the past month, the average visual intensity of the visual static that I experienced was…”), followed by two metrics for symptom *electronic interference* (e.g., “In the past month, the average degree that visual static interfered with my vision while looking at electronic devices (smart phone, tablet, computer, TV screen, etc.) was…” and the next concerning *environment interference*, “In the past month, the average degree that the visual static interfered with my ability to see things in my environment, not related to computer or screen viewing, such as faces, objects, written words on paper, etc. was…”), and finally, one metric for symptom-specific *reduction of daily activities* (e.g., “In the past month, the average degree that the visual static reduced my ability to perform my daily activities was…”). The final four symptoms are non-visual, and therefore use different wording (e.g., tinnitus follows *intensity*, *interference with hearing*, *interference with sleep*, and *reduction of daily activities*). All four symptom metrics were answered with a 1–5 Likert scale. Part 2 of the CVSS is a self-report of symptom change but is not used in the present study. The full CVSS is provided in Appendix A.

The goal of the present investigation was to initiate validation of the CVSS by focusing only on Part 1 (Symptom Assessment) and assessing performance of the CVSS in participants with VSS and controls.

## 2 Materials and methods

### 2.1 Participants

This study was conducted in accordance with the ethical standards set forth in the Declaration of Helsinki and approved by the Institutional Review Board (IRB) of Binghamton University. Informed consent was obtained via Qualtrics from all participants prior to their inclusion in the study.

Control participants were recruited from the Binghamton University research participation pool for credits as part of a degree requirement and participants with VSS were recruited from an advertisement on the Visual Snow Initiative website (5). If a participant reported psychoactive or psychedelic drug use in the past 12 months and had persistent hallucinogenic effects, they were removed from analyses to eliminate overlap with possible Hallucinogen Persisting Perception Disorder (HPPD), since HPPD can mimic VSS (21, 6, 7). Additionally, if a participant declared themself as having VSS, but stated they did not experience visual static, they were removed from the study (as this should not be possible). Note that control participants were informed that the study was directed at VSS; this may have affected recruitment, as participants within a pool self-selected into the study.

The study was administered asynchronously through Qualtrics. Before taking the CVSS, all participants read the ICHD-3 diagnostic criteria, as well as a rendering of visual static, and were asked to self-report if they believed they had VSS. If self-reported VSS, they were additionally asked to self-report if they had been formally diagnosed by a physician. Participants who answered “yes” to this question became our VSS diagnosed (VSS-D) sample, while participants who answered “no” became our VSS undiagnosed (VSS-UD) sample. All other participants were labeled as controls.

Participants were instructed to complete the study alone, in a quiet setting, without distractions. All participants were given a unique, randomly generated URL to the survey. Experimenters were not given access to participant identities beyond the random string generated with each URL. Data was stored on a password-protected university computer. All code and anonymized data is available at Open Science Framework: https://osf.io/x5sru/.

### 2.2 Statistical approach

We sought to identify (1) which symptoms show differences between self-reported severity and impairment, (2) the consistency of responses for each symptom, (3) the symptoms that are most useful for identifying VSS presence.

#### 2.2.1 Between-group comparisons

A series of analysis-of-variance (ANOVA) tests were used to identify differences between three groups: VSS-D (i.e., self-reported physician diagnosis), VSS-UD (i.e., self-diagnosed), and control sample. Then, we used a Tukey’s HSD to record pairwise comparisons. Because we effectively performed 40 different ANOVAs (one for each metric), we opted to set our significance threshold at 𝛼 = 0.05/40 = 0.00125 (i.e., a Bonferroni correction). Additionally, to account for the unequal variances between our VSS samples and control sample, we applied a Greenhouse–Geisser correction to each ANOVA.

#### 2.2.2 Internal consistency

To investigate internal consistency, we evaluated Cronbach’s alpha for the four metrics for each symptom. Specifically, we computed Cronbach’s alpha for each symptom (i.e., across each symptom’s four metrics) for the participants that experienced a given symptom. Following Tavakol & Dennick (8), we set an optimal range for internal consistency at a minimum of 0.7 and maximum of 0.9. This threshold ensured each question captured new information about the same symptom, while still avoiding redundancy.

### 2.3 Principal components of CVSS

To investigate which items on the CVSS best predict group membership (i.e., VSS as compared to not VSS), we conducted a logistic regression and a principal component analysis (PCA).

#### 2.3.1 Logistic regression

We utilized a logistic regression to identify which specific symptoms best predict group membership. Because visual static is required for VSS, we omitted it from our logistic regression model.^1^ Thus, our final generalized linear model (GLM) formula was: group ~ afterimages + trails + BFEP + floaters + nightvision + tinnitus + depersonalization + anxiety + sadness, where each predictor is the average score of each symptom’s four metrics. We computed McFadden’s pseudo *R^2^* with pR2() (9) to quantify how well our GLM fit the data. To compute the odds ratios of each symptom that was used as a predictor, we used coef() to first extract the model coefficients (which are given in log odds), then exp() to convert coefficients into estimated odds ratios.

#### 2.3.2 Principal component analysis

We used a principal component analysis (PCA) to use a data-driven method to cluster which metrics (if any) cluster together. The purpose of this particular analysis, alongside the logistic regression, was to identify if certain symptoms emerged as more predictive than others when predicting group membership. We used prcomp() (10) to identify underlying components accounting for the spread of our data on group-obscured, normalized dataset.

## 3 Results

### 3.1 Participants

A total of 144 participants were recruited, 92 were female (63.9%), with 78 control participants and 66 participants with VSS. We recruited every potential VSS participant who responded to our advertisement posted on the Visual Snow Initiative website (5) if they met the criteria listed above. The control sample was sized to match, but with the caveat that the overall sample should exceed 100. No power analysis was conducted, as the size of the sample was seen as likely to be limited by the recruitment of VSS population participants and little prior work on which to base an estimate of effect size exists, but consideration was given to the approach outlined by Machin et al. (11) in determining the number. One goal of this study was to demonstrate effective differences and provide evidence of effect sizes for future research. The VSS population was divided into two subgroups: those with a self-diagnosis of VSS (“undiagnosed”; VSS-UD) and those with self-report of a VSS diagnosis by a physician (“diagnosed”; VSS-D). Nine participants from the university recruitment pool were included in the VSS sample and placed into the appropriate subgroup as they self-reported visual snow syndrome diagnosis (or self-diagnosis). Eight participants were removed from the sample completely for declaring themselves as having VSS but not experiencing visual static at all, because this symptom is considered to be critical for the diagnosis (per ICHD-3 criteria). Detailed demographics for the full sample are displayed in Table 2.

**Table 2:** Participant demographics, including both control and clinical samples.

	VSS (Total)	VSS-D	VSS-UD	Control	Total
*N*	66	34	32	78	144
Mean age (SD)	37.1 (12.7)	37.0 (12.0)	37.1 (13.7)	18.8 (0.89)	27.2 (12.6)
Gender (Male)	26	17	9	20	46
Gender (Female)	37	16	21	55	92
Gender (Nonbinary)	3	1	2	2	5
Gender (Other)	0	0	0	1	1
Race (White)	56	28	28	48	104
Race (Black)	2	1	1	1	3
Ethnicity (Hispanic)	0	0	1	8	8
Race (Asian/Pacific Islander)	5	3	2	18	23
Race (Native American)	0	0	0	0	0
Race and Ethnicity (Multiple/Other)	3	2	1	3	6

**Figure 1:**
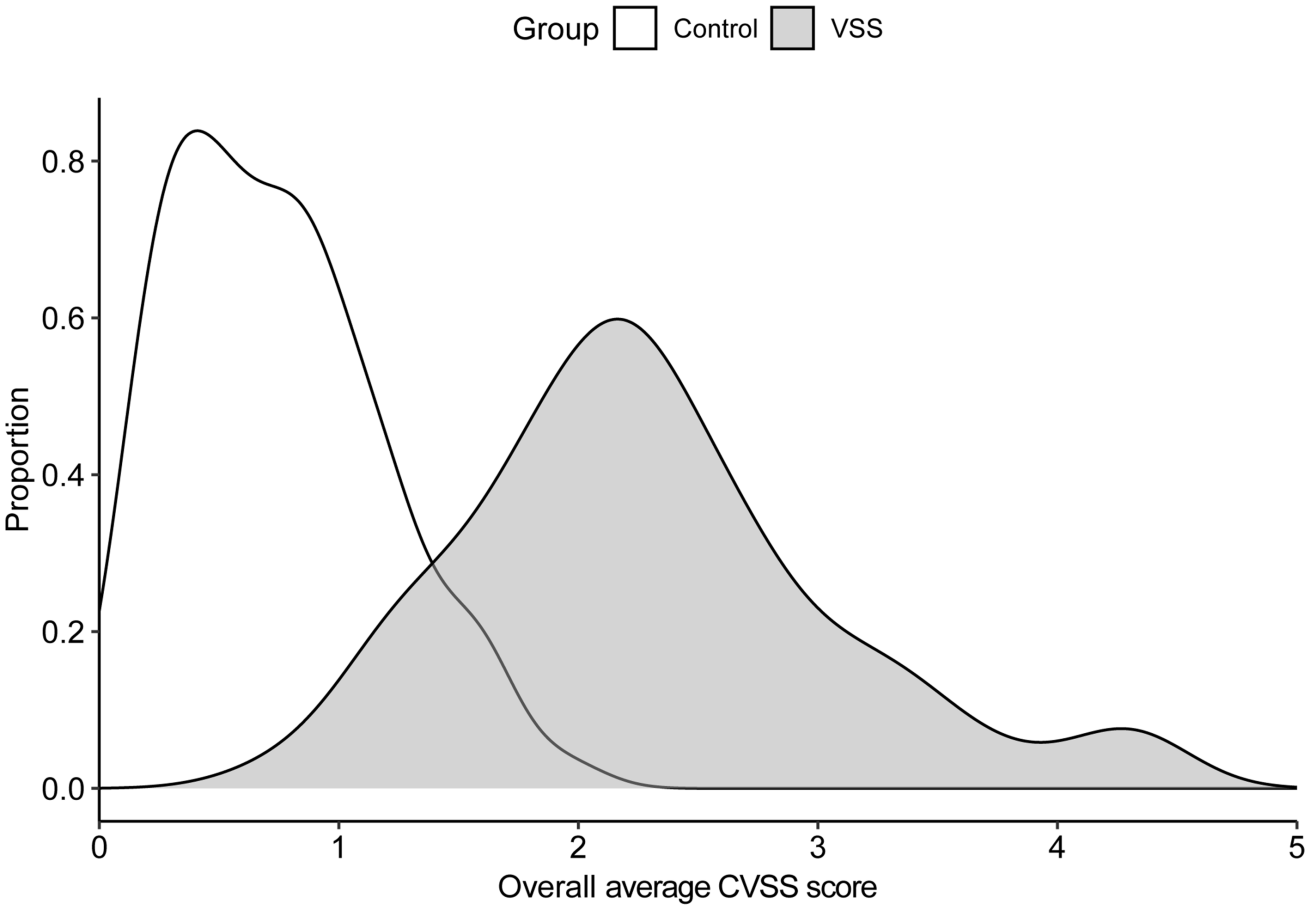
Density of overall CVSS average score by group.

The average Likert score across all metrics for all 10 symptoms for the total VSS group was 2.27 (SD = 0.79); the average for the control group was 0.74 (SD = 0.43). Figure 1 contains the score density (i.e., frequency of response/total responses), across symptoms. Symptom prevalences are listed in Table 3.

**Table 3:** Symptom presence rates, as determined by responses to the CVSS.

	VSS (Total)	VSS (Diagnosed)	VSS (Undiagnosed)	Control
*N* (%)	66	34	32	78
Visual static	66 (100.0%)	34 (100.0%)	32 (100.0%)	2 (2.6%)
Afterimages	58 (87.9%)	32 (94.1%)	26 (81.3%)	15 (19.2%)
Trails	34 (51.5%)	20 (58.8%)	14 (43.8%)	5 (6.4%)
Blue field entoptic phenomenon	53 (80.3%)	27 (79.4%)	26 (81.3%)	16 (20.5%)
Floaters	53 (80.3%)	27 (79.4%)	26 (81.3%)	33 (42.3%)
Diminished night vision	58 (87.9%)	30 (88.2%)	28 (87.5%)	11 (14.1%)
Tinnitus	54 (81.8%)	27 (79.4%)	27 (84.4%)	45 (57.7%)
Depersonalization/derealization	47 (71.2%)	23 (67.6%)	24 (75.0%)	24 (30.8%)
Anxiety	62 (93.9%)	33 (97.1%)	29 (90.6%)	65 (83.3%)
Sadness/loss of interest	50 (75.8%)	24 (70.6%)	26 (81.3%)	45 (57.7%)

### 3.2 Between-group comparisons

**Figure 2:**
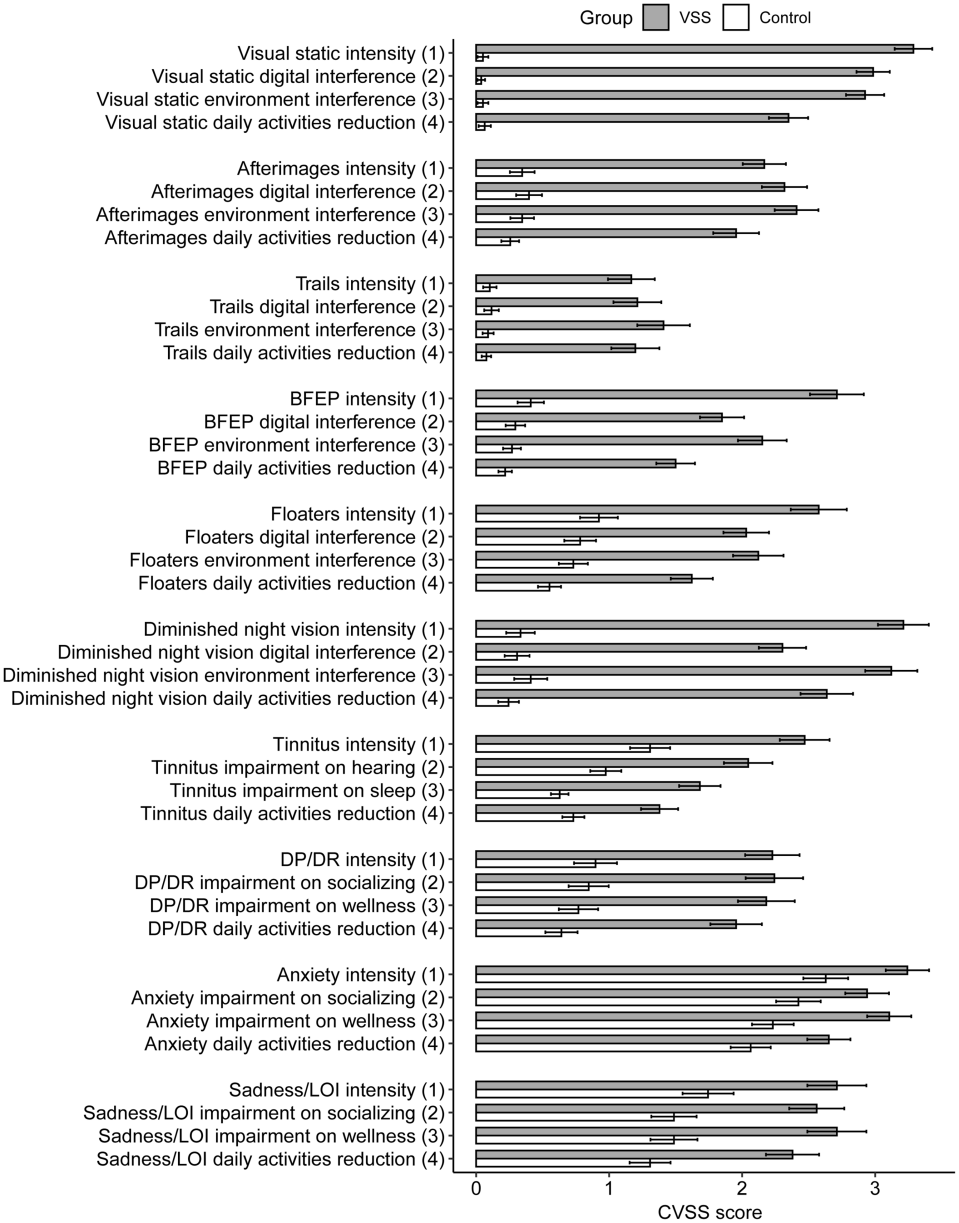
Average Likert score by group and CVSS metric, separated by group membership. BFEP = blue field entoptic phenomenon, DP/DR = depersonalization/derealization, LOI = loss of interest.

**Figure 3:**
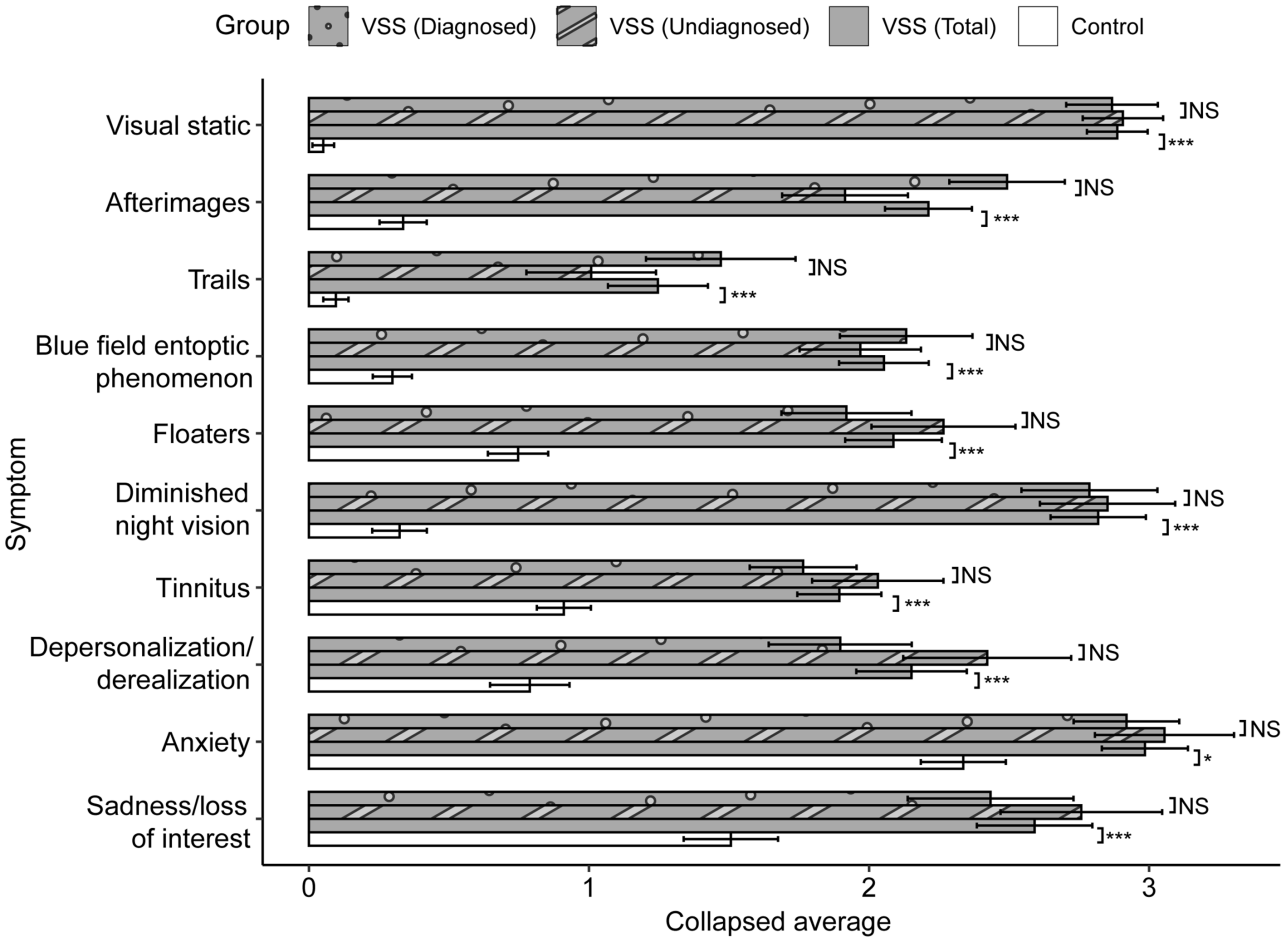
Collapsed between-group comparisons. Scores averaged across the four metrics for each symptom, range from 0 to 5. *p* values displayed after Greenhouse–Geisser correction. After Bonferroni correction, significance threshold is *p* < 0.005. NS: *p* > 0.01; + < 0.01; * < 0.005; ** < 0.001; *** < 0.0001.

The average CVSS scores for 36 out of 40 metrics were significantly greater for the VSS total sample compared to the control group, *p* < 0.05/40, with 32 of 40 metrics withstanding significance at *p* < 0.001/40 (Supplementary Table S1, metric means in Figure 2). The 4 metric average for each symptom demonstrated significant differences in all 10 symptoms between the total VSS sample and the control sample, *p* < 0.05/10, while 0/10 significant differences between the VSS diagnosed and undiagnosed sample (Figure 3). Our diagnosed and undiagnosed VSS samples did not significantly differ for any metric, and will be treated as one sample moving forward.

### 3.3 Internal consistency

To measure internal consistency, we computed the Cronbach’s alpha for each symptom within each group (Table 4), with our optimal range defined between 0.7–0.9. The mean Cronbach’s alpha averaged across groups and all symptoms was *α* = 0.84 (SD = 0.10). In our VSS sample, the metrics pertaining to afterimages, trails, floaters, and anxiety, and sadness symptoms all fell above our optimal range, which indicated these questions may be too similar to one another.

**Table 4:** Cronbach alpha coefficient for Part 1 CVSS symptoms.

Symptom	VSS (Total)	Control
Visual static	0.78	0.96
Afterimages	0.91	0.86
Trails	0.93	0.92
Bluefield entoptic phenomenon	0.85	0.66
Floaters	0.91	0.70
Diminished night vision	0.80	0.83
Tinnitus	0.84	0.56
Depersonalization/derealization	0.87	0.84
Anxiety	0.93	0.90
Sadness/loss of interest	0.90	0.92

### 3.4 Logistic regression analysis

To assess which metrics best predict group membership, we computed a logistic regression using each individual’s average score for 9 symptoms from Part 1 of the CVSS (visual static was omitted, see section 2.3.1) as predictors. Group membership (control vs. VSS) was the predicted variable; status as -D or -UD was not included. We treated our predictor variables as numerics (as opposed to ordered factors).

Ultimately, we found our model–even with visual static omitted–demonstrated a strong fit for our data, McFadden’s pseudo *R^2^* = 0.79. We display below in Table 5 the odds ratios and 95% confidence intervals of each included symptom. In brief, the odds ratio reported indicates *how many times more likely* someone is to have VSS if they display a given symptom, compared to someone who does not display the given symptom.

**Table 5:** Odds ratios by symptom.

Symptom	Odds ratios [95% CI]	*p* value
Afterimages	3.20 [1.27, 9.56]	**0.02**
Trails	2.50 [0.89, 10.48]	0.12
Blue field entoptic phenomenon	5.59 [2.11, 22.16]	**<0.01**
Floaters	0.85 [0.34, 2.01]	0.71
Diminished night vision	3.63 [1.91, 8.88]	**<0.001**
Tinnitus	3.24 [1.13, 11.87]	**0.04**
Depersonalization/derealization	1.89 [0.88, 4.57]	0.11
Anxiety	0.81 [0.44, 1.72]	0.63
Sadness/loss of interest	0.87 [0.30, 1.84]	0.69

Statistically significant results (*p* < 0.05) are bolded.

### 3.5 Principal component analysis

For the 40 total metrics (i.e., 4 metrics on *intensity*, *interference* questions, and *reduction of daily activities* for each of the 10 symptoms), we sought to identify which metrics shared similar patterns of responses through the use of a principal component analysis (PCA). The model generated from the PCA indicated that 56.9% of the variance was from two principal components. Factor 1 was most associated with the metrics from the symptoms: visual static, afterimages, blue field entoptic phenomenon, and night vision impairment, while Factor 2 was most associated with anxiety, sadness, and depersonalization/derealization. This pattern continues even when visual static is removed from the analysis (Supplementary Table S2). For visual clarity, we averaged each symptom’s four metric factor loadings in Table 6.

**Table 6:** Loadings of each CVSS symptom.

Symptom	Factor 1	Factor 2
Visual static	0.20	−0.11
Afterimages	0.19	−0.11
Trails	0.16	−0.04
Blue field entoptic phenomenon	0.18	−0.03
Floaters	0.14	−0.07
Diminished night vision	0.17	−0.15
Tinnitus	0.13	−0.05
Depersonalization/derealization	0.15	0.20
Anxiety	0.11	0.27
Sadness/loss of interest	0.14	0.27

Factor 1 explained 45.1% of variance, while Factor 2 explained with 11.8% of variance.

## 4 Discussion

With this study, we sought to investigate the performance of the CVSS in those with VSS and a control group. Our findings indicate that internal consistency of the CVSS is optimal in 5/10 symptoms (as assessed by responses from the VSS sample), and that the instrument robustly differentiates between those with VSS and a healthy, albeit quite younger, control population. Thus, the CVSS has potential as a novel, patient-reported outcome measure for determining the degree of severity and functional impairment for 10 symptoms that are core features of diagnostic criteria and/or commonly associated with the VSS.

We demonstrated that the majority of metric scores from Part 1 (36 out of 40, 90%) were significantly increased in our total VSS sample in comparison to our control sample (*p* < 0.05/40), with 32 of 40 metrics (80%) in particular withstanding a highly conservative Bonferroni correction (*p* < 0.001/40; Supplementary Table S1). The symptoms that did not significantly differ from our control sample were anxiety metrics (*intensity*, *socializing interference*, and *reduction of daily activities*) and sadness/loss of interest *intensity*. Interestingly, our PCA captured these two symptoms, along with depersonalization/derealization, as most aligning with Factor 2, with the remaining symptoms aligning with Factor 1 (Table 6). These three symptoms are not part of the formal diagnostic criteria for VSS, but we explore how these symptoms connect with a broader picture of mental health in section 4.1. For now, we conclude that our between-group comparison results suggest the CVSS demonstrates high content validity.

Our series of Cronbach’s alpha analyses in the VSS sample demonstrated high internal consistency within all symptoms (i.e., across the four metrics), as all alphas were above 0.70 (Table 4). However, for afterimages, trails, floaters, anxiety, and sadness, there may be some level of redundancy due to the alpha exceeding 0.90 (8). It may be useful for future editions of the CVSS to modify the wordings of these symptoms or consolidate questions for these symptoms altogether.

Our remaining analyses provide evidence that the CVSS can predict VSS and demonstrate which symptoms contribute the most variance to group membership. The logistic regression results suggested that scores pertaining to blue field entoptic phenomena, night vision impairment, tinnitus, and afterimages (in that order) significantly predicted group membership (Table 5). It is particularly interesting to see tinnitus emerge as a significant predictor of group membership, given that the remaining significant symptoms are already ICHD-3 diagnostic criteria (while tinnitus is not). These results, combined with the widespread association of tinnitus with VSS (2, 12, 13), suggest that tinnitus may indeed be a worthwhile symptom to include in future editions of the ICHD diagnostic criteria.

Our findings concerning entoptic phenomena (blue field entoptic phenomenon and floaters) support the idea that people with VSS experience these common phenomena, which are rather prevalent even in a healthy population (Table 3, see (14)) in an ‘enhanced’ manner compared to healthy controls, lending support to the consensus ICHD-3 criteria that enhanced entoptic phenomenon–particularly bluefield entoptic phenomena–is a core feature of VSS. The differential perceptual experience indicated by these outcomes from the CVSS is supported by prior physiologic evidence showing signs of hyperexcitability, such as the lack of habituation in those with VSS compared to controls (15–17). Altogether, these findings provide insight into which symptoms are particularly relevant for diagnosing VSS and for impairing function, and they demonstrate strong support for the ICHD-3 criteria for VSS diagnosis.

### 4.1 Visual snow syndrome and mental health

Even in the earliest discoveries into visual snow syndrome, depression and anxiety were found to be common comorbidities (1). More recently, researchers studying the psychiatric profile of VSS found VSS patients displayed heightened rates of anxiety, depression, and depersonalization, relative to neurotypical controls (6). While our results cannot speak to depression, our results do demonstrate (1) higher prevalence rates of depersonalization, anxiety, and sadness in our VSS sample compared to our control sample (Table 3), a (2) much higher Likert scores on all four sadness/depersonalization metrics (*intensity*, *interference with socializing*, *interference with wellness,* and *reduction of daily activity*) in our VSS sample compared to our control sample (Supplementary Table S1).

Our lack of statistical difference between one sadness and three anxiety metric scores between our VSS sample and control sample may be due to the fact that our VSS sample was significantly older than our control sample, as our control sample was composed primarily of university students, a population known to experience higher self-reported scores of depression than the general population (18). In other words, we compared our VSS sample to a sample that may already experience depression with a higher severity ratings, relative to a true general population. Therefore, we do not discount the possibility that a VSS sample could report higher metric scores of depression and/or anxiety relative to a true general population.

In any case, both our results and previous results speak to a broader mental health concern associated with VSS, and in particular, depersonalization and derealization. We echo the concerns of Solly et al. (6): it is unclear if depersonalization arises out of the same mechanisms that induce VSS, or if the primary symptoms of VSS (i.e., visual static, etc.) give rise to depersonalization. In either case, psychological symptoms–and depersonalization/derealization in particular–deserve additional emphasis in treatment of VSS in order to improve patient quality of life.

### 4.2 Sample representation

Our VSS sample is similar to other VSS samples in the literature in terms of demographics and symptom presence, except the VSS group in this study had a higher prevalence of tinnitus than the group studied by Thompson et al. (7). All the other symptoms were within 10% of the prevalence noted in other studies (1, 2, 7).

### 4.3 Limitations and future research

One limitation for our study is the use of self-reported physician VSS diagnosis and self-diagnosis of VSS and the age differences between the VSS group and the control group. It is possible that members of our VSS-D sample do not actually have a physician diagnosis of VSS, or that people in our VSS sample more broadly do not have VSS.

A second limitation is the stark age difference in ages between our VSS samples (M = 37.1, SD = 12.7) and control sample (M = 18.8, SD = 0.89). In an ideal study, controls would be age-matched to VSS participants. However, it is worth noting that a cursory series of zero-order Spearman correlations revealed that only afterimage scores and sadness/LOI intensity scores correlated with age (Supplementary Table S3). Still, we acknowledge that we cannot rule out the influence of age on our results.

## 5 Conclusion

The CVSS was shown to robustly differentiate participants with VSS and healthy controls. In particular, the degree of night vision impairment, blue field entoptic phenomenon, and afterimages, and tinnitus (in that order) best predicted group membership. We also find support for psychological symptoms, like depersonalization/derealization, as a possible useful therapeutic target to improve patient quality of life. Ultimately, our findings suggest that the CVSS has strong potential as a patient-reported outcome measure for VSS severity and functional impairment in treatment trials.

## Data Availability

The datasets presented in this study can be found in online repositories. The names of the repository/repositories and accession number(s) can be found at: https://osf.io/x5sru/.

## Footnotes

^1^Put another way, the mere absence of visual static perfectly predicted group membership (per our inclusion criteria, which required individuals who identified as VSS to display visual static).
