# Supplementary material for: Assessment of a novel patient reported outcome measure for visual snow syndrome: the Colorado visual snow survey 2.0
Source: Front Neurol. 2025 Sep 29;16:1664310. doi: 10.3389/fneur.2025.1664310 (PMC12515653; doi:10.3389/fneur.2025.1664310)
Supplement: Supplementary file 1 [file Data_Sheet_1.pdf]

CVSS (version 2.0, May 2023). All rights reserved. Email Victoria.Pelak@CUAnschutz.edu for permissions.

Colorado Visual Snow Symptom Scale (CVSS)

Examiner Copy

Note: the CVSS to be given to the participants appears below the examiner copy.

Background and instructions:

The examiner should be as familiar with the CVSS as possible before administering the scale to a participant. It is recommended that you practice with a non-participant 1-2 times to be comfortable with instructions. Use the same instructions with each participant and prevent deviations that might alter the way a participant completes the scale.

The CVSS consists of two parts: 1) assessment of symptoms for the prior month and 2) sense of symptom change during the prior month.

- Part 1 (Symptom Assessment) consists of 10 sections that require a participant to self-evaluate 10 visual snow symptoms that include visual static, visual afterimages, visual trails, blue field entoptic phenomenon, floaters, night vision problems, tinnitus, depersonalization/derealization, anxiety, sadness/loss of interest.
- Part 2 (Sense of Change) requires a participant to self-evaluate change in the 10 symptoms over the past month.

First, the examiner should review with the participant that there are two parts (Part 1 to assess symptoms and Part 2 to assess symptom change – both over the past month). Ask the participant to first briefly review the CVSS without filling in any answers. They should be allowed up to 5 minutes to review the CVSS and become familiar with the format. Remind the participant, as you ask them to review the CVSS, that they should keep in mind that their answers will reflect their *average experience during the past 1 month.*

Next, after the participant reviews the CVSS, ask them if they are ready to begin. Inform them that for each section in Part 1, before they start you will read 1) the introduction, 2) the first question (which asks if they have experienced that symptom), and 3) the definition of terms. Then, if the answer to the first question is yes, (i.e., they have experienced that symptom during the past month), ask them to complete the four questions for that section on their own by circling one choice per question and to let you know when they are done with that section. After they have completed a section, then repeat the process for each section

Once Part 1 is completed explain Part 2. Inform them that Part 2 consists of a table they are to fill out and note to them that for each row, only one choice can be checked.

Before the participant leaves, make sure all questions are answered.

CVSS (version 2.0, May 2023). All rights reserved. Email Victoria.Pelak@CUAnschutz.edu for permissions.

Scoring: There are two main scores for the CVSS.

- **CVSS Total Score**: Part 1 responses create the CVSS Total Score. Part 1 responses can be used to create eight subscores, as explained below.
- **CVSS Sense of Change Score**: Part 2 responses will be used to create the CVSS Sense of Change Score.

Part 1. CVSS Total Score: This score ranges from 4 to 200 points. In Part 1, Sections 2-10 each have a possible score of 0 to 20, while Section 1 must have at least 4 points, since Section 1 concerns visual static, and participants must have visual static to be diagnosed with visual snow syndrome. As noted below, if a participant did not experience the symptom during the past month, then the score for that section is 0. To calculate the score for each section, total all the subsection scores. To calculate the CVSS Total score, total all the section scores.

Part 2. CVSS Sense of Change Score: See the table for Part 2 for further explanation. The participant must choose one of four options for each of the 10 symptoms.

- Option 1 – No change during the prior month: If the participant chooses this option, then the score for that symptom is 0.
- Option 2 – Worsened/Newly Developed/Returned (after being absent) during the prior month: If the participant chooses this option, then the score for that symptom is -1.
- Option 3 – Improved/Stopped (after being present) during the prior month: If the participant chooses this option, then the score for that symptom is +1.
- Option 4 – Never experienced during the prior month: If the participant chooses this option, then the score for that symptom is 0. To calculate the CVSS Sense of Change Score, add each symptom change score to find the CVSS Sense of Change Score.

Part 1 CVSS subscores: There are 8 subscores, which include the following: intensity, reduction of daily activities, interference with vision on electronic devices, interference with vision in the environment, tinnitus interference with hearing, tinnitus interference with sleep, interference with socializing, and interference with feelings of wellness.

- In Part 1, each of the 10 sections have subsections on intensity and reduction of daily activities, and the following two subscores using results from the entire scale can determined as follows*:*

  1. Intensity subscore: Add the scores from each of the "A Subsections" in Sections 1-10; the range for this subscore is 1-50.

  2. Reduction of daily activities subscore: Add the scores from each of the "D Subsections" in Sections 1-10; the range for this subscore is 1-50.

CVSS (version 2.0, May 2023). All rights reserved. Email Victoria.Pelak@CUAnschutz.edu for permissions.

- In Part 1, each of the 10 sections has two subsections regarding symptom interference, and the type of interference varies by symptom, which gives the following subscores:

  3. Interference with vision on electronic devices subscore: Add the scores from the "B Subsections" in Sections 1-6; the range for this subscore 1-30.

  4. Interference with vision in the environment subscore: Add the scores from the "C Subsections" in Sections 1-6; the range for this subscore is 1-30.

  5. Tinnitus interference with hearing subscore: Use the score from the "B Subsection" in Section 7 only; the range for this subscore is 0-5.

  6. Tinnitus interference with sleep subscore: Use the score from the "C Subsection" in Section 7 only; the range for this subscore is 0-5.

  7. Interference with socializing subscore: Add the scores from the "B Subsections" in Sections 8-10; the range for this subscore is 0-15.

  8. Interference with feelings of wellness subscore: Add the scores from the "C Subsections" in Sections 8-10; the range for this subscore is 0-15.

CVSS (version 2.0, May 2023). All rights reserved. Email Victoria.Pelak@CUAnschutz.edu for permissions.

Examiner Copy of the CVSS PART 1.

Section 1. Visual Static.

Examiner to read the following: Section 1 has four statements concerning how you have experienced visual static over the past month. Please answer by considering what your average experience has been over the past month. You can now review the statements and circle one response for each of the four statements.

A. Visual Intensity of Static: In the past month, the **average** visual intensity of the **visual static** that I experienced was

1	2	3	4	5
Not intense<br><br>(static was not distinct and is muted or dim)	Weakly intense	Mildly intense<br><br>(static was distinct but not vivid or bright)	Moderately intense	Severely intense<br><br>(static was distinct and was vivid or bright)

B. Interference with vision while viewing electronic devices: In the past month, the **average** degree that **visual static** interfered with my vision while looking at electronic devices (smart phone, tablet, computer, TV screen, etc.) was

1	2	3	4	5
No interference<br><br>(able see clearly past the static)	Weak interference	Mild interference<br><br>(most of the time I was able to see past the static but with mild difficulty)	Moderate interference	Severe interference<br><br>(never able to see clearly past the static)

CVSS (version 2.0, May 2023). All rights reserved. Email Victoria.Pelak@CUAnschutz.edu for permissions.

C. Interference with vision while viewing the environment: In the past month, the **average** degree that the **visual static** interfered with my ability to see things in my environment, **not** related to computer or screen viewing, such as faces, objects, written words on paper, etc. was

1	2	3	4	5
No interference<br><br>(able see clearly past the static)	Weak interference	Mild interference<br><br>(most of the time able to see past the static but with mild difficulty)	Moderate interference	Severe interference<br><br>(never able to see clearly past the static)

D. Reduction of daily activities: In the past month, the **average** degree that the **visual static** reduced my ability to perform my daily activities was

1	2	3	4	5
No reduction<br><br>(the static never reduced my ability to perform activities at any time)	Minimal reduction<br><br>(the static reduced my ability to perform activities between 1% and 25% of the time)	Mild reduction<br><br>(the static reduced my ability to perform activities greater than 25% of the time but less than 50% of the time)	Moderate reduction<br><br>(the static reduced my ability to perform activities greater than 50% of the time but less than 75% of the time)	Severe reduction<br><br>(the static reduced my ability to perform activities greater than 75% of the time)

Section 2. Afterimages.

Examiner to read the following: Section 2 concerns afterimages. You will be asked if you have experienced afterimages in the past month, and if you have not, circle no and we will move on to Section 3. If you have, then you will complete four statements concerning how you have experienced afterimages over the past month. Please answer by considering what your average experience has been over the past month. I will read the definitions and you can then circle your responses for each of the four statements if you have experienced afterimages in the past month.

CVSS (version 2.0, May 2023). All rights reserved. Email Victoria.Pelak@CUAnschutz.edu for permissions.

Definitions

- **Negative visual afterimage:** after shifting gaze away from viewing an object, there is a continued perception of the object appearing as the opposite color of the object.
- **Positive visual afterimage:** after shifting gaze away from viewing an object, there is a continued perception of the object appearing as the same color as the object.

I have experienced seeing positive and/or negative afterimages in the past month: Yes or No (if no, move to next section)

A. Intensity of Negative and/or **Positive Afterimages**: In the past month, the **average** visual intensity of the **afterimages** I experienced was

1	2	3	4	5
Not intense<br><br>(never vivid and lasted **less than** 60 seconds if negative **and less than** 1 second if positive)	Minimally intense<br><br>(vivid at times **and** lasted **less than** 60 seconds if negative and **less than** 1 second if positive)	Mildly intense<br><br>(vivid at times and lasted **greater than** 60 seconds if negative or **greater than** 1 second if positive)	Moderately intense<br><br>(vivid in most instances and lasted **greater than** 60 seconds if negative or **greater than** 1 second if positive)	Severely intense<br><br>(vivid in all instances and lasted **greater than** 60 seconds if negative or **greater than** 1 second if positive)

B. Interference with vision while viewing electronic devices: In the past month, the **average** degree that the **afterimages** interfered with my vision after looking at electronic devices (computer or TV screen) was

1	2	3	4	5
No interference<br><br>(able see clearly during the afterimages without difficulty)	Minimal interference<br><br>(able to see during the afterimages with minimal difficulty)	Mild interference<br><br>(able to see during the afterimages with mild difficulty)	Moderate interference<br><br>(able to see during the afterimages with moderate difficulty)	Severe interference<br><br>(never able to see clearly during the afterimages)

C. Interference with vision while viewing the environment: In the past month, the **average** degree that the **afterimages** interfered with my ability to see things in my environment, **not** related to electronic devices, such as faces, objects, written words on paper, etc., was

CVSS (version 2.0, May 2023). All rights reserved. Email Victoria.Pelak@CUAnschutz.edu for permissions.

1	2	3	4	5
No interference<br><br>(able see clearly during the afterimages without difficulty)	Weak interference	Mild interference<br><br>(able to see during the afterimages but with mild difficulty)	Moderate interference	Severe interference<br><br>(never able to see clearly during the afterimages)

D. Reduction of daily activities: In the past month, the **average** degree that the **afterimages** reduced my ability to perform my daily activities was

1	2	3	4	5
No reduction<br><br>(the afterimages never reduced my ability to perform activities at any time)	Minimal reduction<br><br>(the afterimages reduced my ability to perform activities between 1% and 25% of the time)	Mild reduction<br><br>(the afterimages reduced my ability to perform activities greater than 25% of the time but less than 50% of the time)	Moderate reduction<br><br>(the afterimages reduced my ability to perform activities greater than 50% of the time but less than 75% of the time)	Severe reduction<br><br>(the afterimages reduced my ability to perform activities greater than 75% of the time)

Section 3. Trails.

Examiner to read the following: Section 3 concerns trails. You will be asked if you have experienced trails in the past month, and if you have not, circle no, and we will move on to Section 4. If you have, then you will complete four statements concerning how you have experienced visual trails over the past month. Please answer by considering what your average experience has been over the past month. I will read the definitions and you can then circle your responses for each of the four statements if you have experienced trails in the past month.

Definitions:

- **Visual Trails:** While viewing an object in motion, there is a series of images of the object that follow the object as it moves and then the images fade out along the object's path.

I have experienced seeing visual trails in the last month: Yes or No (if no, move to next section)

A. Intensity of Trails: In the past month, the **average** visual intensity of the **trails** that I experienced was

CVSS (version 2.0, May 2023). All rights reserved. Email Victoria.Pelak@CUAnschutz.edu for permissions.

1	2	3	4	5
Not intense<br><br>(the trails were never vivid **and/or** always less than 10 seconds)	Minimally intense<br><br>(in a few instances, the trails were vivid **and** greater than 10 seconds)	Mildly intense<br><br>(as often as not, the trails were vivid **and** greater than 10 seconds)	Moderately intense<br><br>(in most instances, the trails were vivid **and** greater than 10 seconds)	Severely intense<br><br>(in all instances, the trails were vivid **and** greater than 10 seconds)

B. Interference with vision while viewing electronic devices: In the past month, the **<u>average</u>** degree that the **trails** interfered with my vision while using electronic devices (computer or TV screen) was

1	2	3	4	5
No interference<br><br>(able see clearly with trails without difficulty)	Minimal interference<br><br>(able to see during the trails and with minimal difficulty)	Mild interference<br><br>(able to see during the trails but with mild difficulty)	Moderate interference<br><br>(able to see during the trails but with moderate difficulty)	Severe interference<br><br>(never able to see clearly during the trails)

C. Interference with vision while viewing the environment: In the past month, the **<u>average</u>** degree that the **trails** interfered with my ability to see things in my environment, **<u>not</u>** related to electronic devices, such as faces, objects, written words on paper, etc., was

1	2	3	4	5
No interference<br><br>(able see clearly regardless of trails)	Weak interference	Mild interference<br><br>(able to see during the trails but with mild difficulty)	Moderate interference	Severe interference<br><br>(never able to see clearly during the trails)

D. Reduction of daily activities: In the past month, the **<u>average</u>** degree that the **trails** reduced my ability to perform my daily activities was

1	2	3	4	5

CVSS (version 2.0, May 2023). All rights reserved. Email Victoria.Pelak@CUAnschutz.edu for permissions.

No reduction<br><br>(the trails never reduced my ability to perform activities at any time)	Minimal reduction<br><br>(the trails reduced my ability to perform activities between 1% and 25% of the time)	Mild reduction<br><br>(the trails reduced my ability to perform activities greater than 25% of the time but less than 50% of the time)	Moderate reduction<br><br>(the trails reduced my ability to perform activities greater than 50% of the time but less than 75% of the time)	Severe reduction<br><br>(the trails reduced my ability to perform activities greater than 75% of the time)

Section 4. Blue field entoptic phenomenon.

Examiner to read the following: Section 4 concerns blue field entoptic phenomenon. You will be asked if you have experienced this phenomenon in the past month, and if you have not, circle no, and we will move on to Section 5. If you have, then you will complete four statements concerning how you have experienced the phenomenon over the past month. Please answer by considering what your average experience has been over the past month. I will read the definitions and you can then circle your responses for each of the four statements if you have experienced the phenomenon in the past month.

Definitions:

- **Blue field entoptic phenomenon**: While looking at a bright blue background like the sky, small bright dots appear and move quickly along linear or snaking paths in the visual field.

I have experienced blue field entoptic phenomenon in the past month: Yes or No (if no, move to next section)

A. Visual Intensity: In the past month, the **average** visual intensity of the **blue field entoptic phenomenon** that I experienced was

1	2	3	4	5
Not intense<br><br>(the phenomenon was not distinct and was muted or dim)	Weakly intense	Mildly intense<br><br>(the phenomenon was distinct but not vivid or bright)	Moderately intense	Severely intense<br><br>(the phenomenon was distinct and is vivid or bright)

B. Interference with vision while viewing electronic devices: In the past month, the **average** degree that **blue field entoptic phenomenon** interfered with my vision while looking at electronic devices (smart phone, tablet, computer, TV screen, etc.) was

CVSS (version 2.0, May 2023). All rights reserved. Email Victoria.Pelak@CUAnschutz.edu for permissions.

1	2	3	4	5
No interference<br><br>(able see clearly past the phenomenon)	Weak interference	Mild interference<br><br>(able to see past the phenomenon but with mild difficulty)	Moderate interference	Severe interference<br><br>(never able to see clearly past the phenomenon)

C. Interference with vision while viewing the environment: In the past month, the **average** degree that the **blue field entoptic phenomenon** interfered with my ability to see things in my environment, **not** related to electronic devices, such as faces, objects, written words on paper, etc., was

1	2	3	4	5
No interference<br><br>(able see clearly past the phenomenon with no difficulty)	Weak interference	Mild interference<br><br>(able to see past the phenomenon but with mild difficulty)	Moderate interference	Severe interference<br><br>(never able to see clearly past the phenomenon)

D. Reduction of daily activities: In the past month, the **average** degree that the **blue field entoptic phenomenon** reduced my ability to perform my daily activities was

1	2	3	4	5
No reduction<br><br>(the phenomenon never reduced my ability to perform activities at any time)	Minimal reduction<br><br>(the phenomenon reduced my ability to perform activities between 1% and 25% of the time)	Mild reduction<br><br>(the phenomenon reduced my ability to perform activities greater than 25% of the time but less than 50% of the time)	Moderate reduction<br><br>(the phenomenon reduced my ability to perform activities greater than 50% of the time but less than 75% of the time)	Severe reduction<br><br>(the phenomenon reduced my ability to perform activities greater than 75% of the time)

Section 5. Floaters.

Examiner to read the following: Section 5 concerns floaters. You will be asked if you have experienced floaters in the past month, and if you have not, circle no and we will move on to

CVSS (version 2.0, May 2023). All rights reserved. Email Victoria.Pelak@CUAnschutz.edu for permissions.

Section 6. If you have, then you will complete four statements concerning how you have experienced floaters over the past month. Please answer by considering what your average experience has been over the past month. I will read the definitions and you can then circle your responses for each of the four statements if you have experienced floaters in the past month.

Definitions:

- **Floaters**: small, dark or semi-transparent specks, spots, or dots; floaters can also appear as thread-like or cobweb-like shapes; floaters are seen from within the eye and float or drift with your eye movements.

I have experienced seeing floaters in the past month: Yes or No (if no, move to next section)

A. Visual Intensity: In the past month, the **average** visual intensity of the **floaters** that I experienced was

1	2	3	4	5
Not intense<br><br>(the floaters were not distinct, not numerous, and easy to ignore)	Weakly intense	Mildly intense<br><br>(most of the time the floaters were distinct, but not numerous, and could be ignored)	Moderately intense	Severely intense<br><br>(the floaters were distinct, very numerous, and could not be ignored)

B. Interference with vision while viewing electronic devices: In the past month, the **average** degree that **floaters** interfered with my vision while looking at electronic devices (smart phone, tablet, computer, TV screen, etc.) was

1	2	3	4	5
No interference<br><br>(able see clearly past the floaters)	Weak interference	Mild interference<br><br>(able to see past the floaters but with mild difficulty)	Moderate interference	Severe interference<br><br>(never able to see clearly past the floaters)

C. Interference with vision while viewing the environment In the past month, the **average** degree that the **floaters** interfered with my ability to see things in my environment, **not** related to electronic devices, such as faces, objects, written words on paper, etc., was

1	2	3	4	5

CVSS (version 2.0, May 2023). All rights reserved. Email Victoria.Pelak@CUAnschutz.edu for permissions.

No interference<br><br>(able see clearly past the floaters)	Weak interference	Mild interference<br><br>(able to see past the floaters but with mild difficulty)	Moderate interference	Severe interference<br><br>(never able to see clearly past the floaters)

D. Reduction of daily activities: In the past month, the **average** degree that the **floaters** reduced my ability to perform my daily activities was

1	2	3	4	5
No reduction<br><br>(the floaters never reduced my ability to perform activities at any time)	Minimal reduction<br><br>(the floaters reduced my ability to perform activities between 1% and 25% of the time)	Mild reduction<br><br>(the floaters reduced my ability to perform activities greater than 25% of the time but less than 50% of the time)	Moderate reduction<br><br>(the floaters reduced my ability to perform activities greater than 50% of the time but less than 75% of the time)	Severe reduction<br><br>(the floaters reduced my ability to perform activities greater than 75% of the time)

Section 6. Night vision problems.

Examiner to read the following: Section 6 concerns night vision problems. You will be asked if you have experienced problems with your night vision in the past month, and if you have not, circle no and we will move on to Section 7. If you have, then you will complete four statements concerning how you have experienced night vision over the past month. Please answer by considering what your average experience has been over the past month. I will read the definitions and you can then circle your responses for each of the four statements if you have experienced night vision problems in the past month.

Definition:

- **Night vision problems:** Problems with vision at night that started **WITH** or **AFTER** the visual snow or static symptoms started.

I have experienced night vision problems in the past month: Yes or No (if no, move to next section)

A. Overall Intensity: In the past month, the **average** intensity of the **night vision problems** that I have experienced was

1	2	3	4	5

CVSS (version 2.0, May 2023). All rights reserved. Email Victoria.Pelak@CUAnschutz.edu for permissions.

Not intense<br><br>(not noticeable **and/or** can adjust to dark immediately)	Weakly intense	Mildly intense<br><br>(most of the time I was able to ignore the night vision problems **and/or** adjust to dark with mild difficulty)	Moderately intense	Severely intense<br><br>(never able to ignore the night vision problems **and/or** adjust to the dark)

B. Interference with vision while viewing electronic devices: In the past month, the **<u>average</u>** degree that **night vision problems** interfered with my vision while looking at electronic devices (smart phone, tablet, computer, TV screen, etc.) was

1	2	3	4	5
No interference<br><br>(able see clearly at night on a device)	Weak interference	Mild interference<br><br>(most of the time I was able to see clearly at night on a device but with mild difficulty)	Moderate interference	Severe interference<br><br>(never able to see clearly at night on a device)

C. Interference with vision while viewing the environment: In the past month, the **<u>average</u>** degree that the **night vision problems** interfered with my ability to see things in my environment, <u>not</u> related to electronic devices, such as faces, objects, written words on paper, etc., was

1	2	3	4	5
No interference<br><br>(able see clearly at night)	Weak interference	Mild interference<br><br>(most of the time I was able to see at night but with mild difficulty)	Moderate interference	Severe interference<br><br>(never able to see clearly at night)

D. Reduction of daily (nighttime) activities: In the past month, the **<u>average</u>** degree that the **night vision problems** reduced my ability to perform activities at night was

1	2	3	4	5
No reduction	Minimal reduction	Mild reduction	Moderate reduction	Severe reduction

CVSS (version 2.0, May 2023). All rights reserved. Email Victoria.Pelak@CUAnschutz.edu for permissions.

(the night vision issues never reduced my ability to perform activities at any time)	(the night vision issues reduced my ability to perform activities between 1% and 25% of the time)	(the night vision issues reduced my ability to perform activities greater than 25% of the time but less than 50% of the time)	(the night vision issues reduced my ability to perform activities greater than 50% of the time but less than 75% of the time)	(the night vision issues reduced my ability to perform activities greater than 75% of the time)

Section 7. Ringing in the ears or Tinnitus.

Examiner to read the following: Section 7 concerns tinnitus. You will be asked if you have experienced tinnitus in the past month, and if you have not, circle no and we will move on to Section 8. If you have, then you will complete four statements concerning how you have experienced tinnitus over the past month. Please answer by considering what your average experience has been over the past month. I will read the definitions and you can then circle your responses for each of the four statements if you have experienced tinnitus in the past month.

Definition:

- **Tinnitus:** The sound of ringing or buzzing in the head and/or in one or both ears without an external source.

I have experienced tinnitus in the past month: Yes or No (if no, move to next section)

A. Overall Intensity: In the past month, the **average** intensity of the **tinnitus** that I experienced was

**1**	**2**	**3**	**4**	**5**
Not intense<br><br>(not noticeable **and/or** I adjusted immediately)	Weakly intense	Mildly intense<br><br>(most of the time I was able to ignore **and/or** adjust with only mild difficulty)	Moderately intense	Severely intense<br><br>(never able to ignore **and/or** adjust)

B. Interference with hearing: In the past month, the **average** degree that the **tinnitus** interfered with my ability to hear was

**1**	**2**	**3**	**4**	**5**
No interference	Weak interference	Mild interference	Moderate interference	Severe interference

CVSS (version 2.0, May 2023). All rights reserved. Email Victoria.Pelak@CUAnschutz.edu for permissions.

(not noticeable **and/or** I adjusted immediately)		(most of the time I was able to ignore **and/or** adjust with only mild difficulty)		(never able to ignore **and/or** adjust)

C. Interference with sleep: In the past month, the **average** degree that the **tinnitus** interfered with my sleep was

1	2	3	4	5
No interference<br><br>(never interfered my sleep)	Minimal interference<br><br>(interfered with my sleep up to 25% of the time)	Mild interference<br><br>(interfered with my sleep greater than 25% and less than 50% of the time and/or degree)	Moderate interference<br><br>(interfered my sleep between 50-75% of the time)	Severe interference<br><br>(interfered with my sleep greater than 75% of the time)

D. Reduction of daily activities: In the past month, the **average** degree that that the **tinnitus** reduced my ability to perform my daily activities was

1	2	3	4	5
No reduction<br><br>(never reduced my ability to perform activities at any time)	Minimal reduction<br><br>(reduced my ability to perform activities between 1% and 25% of the time)	Mild reduction<br><br>(reduced my ability to perform activities greater than 25% of the time but less than 50% of the time)	Moderate reduction<br><br>(reduced my ability to perform activities greater than 50% of the time but less than 75% of the time)	Severe reduction<br><br>(reduced my ability to perform activities greater than 75% of the time)

Section 8. Depersonalization and derealization.

Examiner to read the following: Section 8 concerns depersonalization and derealization. You will be asked if you have experienced depersonalization and derealization in the past month, and if you have not, circle no and we will move on to Section 9. If you have, then you will complete four statements concerning how you have experienced depersonalization and derealization over the

CVSS (version 2.0, May 2023). All rights reserved. Email Victoria.Pelak@CUAnschutz.edu for permissions.

past month. Please answer by considering what your average experience has been over the past month. I will read the definitions and you can then circle your responses for each of the four statements if you have experienced depersonalization and derealization in the past month.

Definition:

- **Depersonalization**: The feeling of being detached or separate from your thoughts, feelings, and body.
- **Derealization**: The feeling of being disconnected from your environment.
- **Wellness**: The feeling or quality of being in a good state of health.

I have experienced depersonalization or derealization in the past month: Yes or No (if no, move to next section)

A. Overall Intensity: In the past month, the **average** intensity of the **feeling of depersonalization or derealization** that I experienced was

1	2	3	4	5
Not intense<br><br>(not noticeable **and/or** can adjust immediately)	Weakly intense	Mildly intense<br><br>(most of the time I was able to ignore **and/or** adjust with mild difficulty)	Moderately intense	Severely intense<br><br>(never able to ignore **and/or** adjust)

B. Interference with socializing: In the past month, the **average** degree that the **feelings of depersonalization or derealization** interfered with my ability to socialize was

1	2	3	4	5
No interference<br><br>(not noticeable **and/or** I adjusted immediately)	Weak interference	Mild interference<br><br>(most of the time I was able to ignore **and/or** adjust with only mild difficulty)	Moderate interference	Severe interference<br><br>(never able to ignore **and/or** adjust)

C. Interference with feelings of wellness. In the past month, the **average** degree that the feelings of **depersonalization or derealization** interfered with my feeling of wellness was

1	2	3	4	5

CVSS (version 2.0, May 2023). All rights reserved. Email Victoria.Pelak@CUAnschutz.edu for permissions.

No interference<br><br>(not noticeable **and/or** did not impact my wellness)	Minimal interference<br><br>(interfered with my wellness up to 25% of the time)	Mild interference<br><br>(interfered with my wellness greater than 25% and less than 50% of the time)	Moderate interference<br><br>(interfered with my wellness between 50-75% of the time)	Severe interference<br><br>(interfered with my wellness greater than 75% of the time)

D. Reduction of daily activities: In the past month, the **average** degree that that the **depersonalization or derealization** reduced my ability to perform my daily activities was

1	2	3	4	5
No reduction<br><br>(never reduced my ability to perform activities at any time)	Minimal reduction<br><br>(reduced my ability to perform activities between 1% and 25% of the time	Mild reduction<br><br>(reduced my ability to perform activities greater than 25% of the time but less than 50% of the time)	Moderate reduction<br><br>(reduced my ability to perform activities greater than 50% of the time but less than 75% of the time)	Severe reduction<br><br>(reduced my ability to perform activities greater than 75% of the time)

Section 9. Anxiety.

Examiner to read the following: Section 9 concerns anxiety. You will be asked if you have experienced anxiety in the past month, and if you have not, circle no and we will move on to Section 10. If you have, then you will complete four statements concerning how you have experienced anxiety over the past month. Please answer by considering what your average experience has been over the past month. I will read the definitions and you can then circle your responses for each of the four statements if you have experienced anxiety in the past month.

Definition:

- **Anxiety**: Anxiety is uncontrollable feelings of anxiety, fear, worry, and/or panic.
- **Wellness**: The feeling or quality of being in a good state of health.

I have experienced anxiety in the past month: Yes or No (if no, move to next section)

A. Overall Intensity: In the past month, the **average** intensity of **anxiety** that I experienced was

1	2	3	4	5

CVSS (version 2.0, May 2023). All rights reserved. Email Victoria.Pelak@CUAnschutz.edu for permissions.

Not intense<br><br>(not noticeable **and/or** can adjust immediately)	Weakly intense	Mildly intense<br><br>(most of the time I was able to ignore **and/or** adjust with mild difficulty)	Moderately intense	Severely intense<br><br>(never able to ignore **and/or** adjust)

B. Interference with socializing: In the past month, the **<u>average</u>** degree that **anxiety** interfered with my ability to socialize was

**1**	**2**	**3**	**4**	**5**
No interference<br><br>(not noticeable **and/or** I adjusted immediately)	Weak interference	Mild interference<br><br>(most of the time I was able to ignore **and/or** adjust with only mild difficulty)	Moderate interference	Severe interference<br><br>(never able to ignore **and/or** adjust)

C. Interference with feelings of wellness. In the past month, the **<u>average</u>** degree that **anxiety** interfered with my feeling of wellness:

**1**	**2**	**3**	**4**	**5**
No interference<br><br>(not noticeable **and/or** did not impact my wellness)	Minimal interference<br><br>(interfered with my wellness up to 25% of the time)	Mild interference<br><br>(interfered with my wellness greater than 25% and less than 50% of the time)	Moderate interference<br><br>(interfered with my wellness between 50-75% of the time)	Severe interference<br><br>(interfered with my wellness greater than 75% of the time)

CVSS (version 2.0, May 2023). All rights reserved. Email Victoria.Pelak@CUAnschutz.edu for permissions.

D. Reduction of daily activities: In the past month, the **average** degree that **anxiety** reduced my ability to perform my daily activities was

1	2	3	4	5
No reduction<br><br>(never reduced my ability to perform activities at any time)	Minimal reduction<br><br>(reduced my ability to perform activities between 1% and 25% of the time)	Mild reduction<br><br>(reduced my ability to perform activities greater than 25% of the time but less than 50% of the time)	Moderate reduction<br><br>(reduced my ability to perform activities greater than 50% of the time but less than 75% of the time)	Severe reduction<br><br>(reduced my ability to perform activities greater than 75% of the time)

Section 10. Symptoms of sadness or loss of interest.

Examiner to read the following: Section 10 concerns symptoms of sadness or loss of interest. You will be asked if you have experienced sadness or loss of interest in the past month, and if you have not, circle no and we will move on to Part 2. If you have, then you will complete four statements concerning how you have experienced sadness or loss of interest over the past month. Please answer by considering what your average experience has been over the past month. I will read the definitions and you can then circle your responses for each of the four statements if you have experienced sadness or loss of interest in the past month.

Definitions:

- **Sadness**: A feeling of being unhappy.
- **Loss of interest:** No longer interested in activities that you usually find enjoyable.

I have experienced symptoms of sadness or loss of interest in the past month: Yes or No (if no, move to next section)

A. Overall Intensity: In the past month, the **average** intensity **of sadness or loss of interest** that I experienced was

1	2	3	4	5
Not intense<br><br>(not noticeable **and/or** can adjust immediately)	Weakly intense	Mildly intense<br><br>(most of the time I was able to ignore **and/or** adjust with mild difficulty)	Moderately intense	Severely intense<br><br>(never able to ignore **and/or** adjust)

CVSS (version 2.0, May 2023). All rights reserved. Email Victoria.Pelak@CUAnschutz.edu for permissions.

D. **Interference with socializing**: In the past month, the **average** degree that **sadness or loss of interest** interfered with my ability to socialize was

1	2	3	4	5
No interference<br><br>(not noticeable **and/or** I adjusted immediately)	Weak interference	Mild interference<br><br>(most of the time I was able to ignore **and/or** adjust with only mild difficulty)	Moderate interference	Severe interference<br><br>(never able to ignore **and/or** adjust)

C. Interference with feelings of wellness. In the past month, the **average** degree **that sadness or loss of interest** interfered with my feeling of wellness was

1	2	3	4	5
No interference<br><br>(not noticeable **and/or** did not impact my wellness)	Minimal interference<br><br>(interfered with my wellness up to 25% of the time)	Mild interference<br><br>(interfered with my wellness greater than 25% and less than 50% of the time)	Moderate interference<br><br>(interfered with my wellness between 50-75% of the time)	Severe interference<br><br>(interfered with my wellness greater than 75% of the time)

D. Reduction of daily activities: In the past month, the **average** degree that that **sadness or loss of interest** reduced my ability to perform my daily activities was

1	2	3	4	5
No reduction<br><br>(never reduced my ability to perform activities at any time)	Minimal reduction<br><br>(reduced my ability to perform activities between 1% and 25% of the time)	Mild reduction<br><br>(reduced my ability to perform activities greater than 25% of the time but less than 50% of the time)	Moderate reduction<br><br>(reduced my ability to perform activities greater than 50% of the time but less than 75% of the time)	Severe reduction<br><br>(reduced my ability to perform activities greater than 75% of the time)

CVSS (version 2.0, May 2023). All rights reserved. Email Victoria.Pelak@CUAnschutz.edu for permissions.

Examiner Copy CVSS PART 2.

CVSS Sense of Change over the past month

Examiner to read: Now we are ready to move on to Part 2. You will complete a table concerning how your symptoms have changed over the past month. For each row (i.e., each symptom), please place a check in only one of the four columns.

**Symptoms**	**Symptom worsened or newly developed or returned during the past month after being absent** (-1 point will be given for each answer in this column)	**Symptom did not change during the past month** (0 points will be given for each answer in this column)	**Symptom improved or stopped during the past month after being present** (+1 point for each answer in this column)	**Never had this symptom during the past month** (0 points for each answer in this column)
Visual static				Not a choice
Afterimages				
Trails				
Blue field entoptic phenomenon				
Floaters				
Night vision problems				
Tinnitus				
Depersonalization or derealization				
Anxiety				
Sadness or loss of interest				

CVSS (version 2.0, May 2023). All rights reserved. Email Victoria.Pelak@CUAnschutz.edu for permissions.

THIS PAGE LEFT INTENTIONALLY BLANK

CVSS (version 2.0, May 2023). All rights reserved. Email Victoria.Pelak@CUAnschutz.edu for permissions.

Colorado Visual Snow Symptom Scale (CVSS)

Before filling out each section, please wait for instructions.

Section 1. Visual Static.

A. Visual Intensity of Static: In the past month, the **average** visual intensity of the **visual static** that I experienced was

1	2	3	4	5
Not intense<br><br>(static was not distinct and is muted or dim)	Weakly intense	Mildly intense<br><br>(static was distinct but not vivid or bright)	Moderately intense	Severely intense<br><br>(static was distinct and was vivid or bright)

B. Interference with vision while viewing electronic devices: In the past month, the **average** degree that the **visual static** interfered with my vision while looking at electronic devices (smart phone, tablet, computer, TV screen, etc.) was

1	2	3	4	5
No interference<br><br>(able see clearly past the static)	Weak interference	Mild interference<br><br>(most of the time I was able to see past the static but with mild difficulty)	Moderate interference	Severe interference<br><br>(never able to see clearly past the static)

C. Interference with vision while viewing the environment: In the past month, the **average** degree that the **visual static** interfered with my ability to see things in my environment, **not** related to computer or screen viewing, such as faces, objects, written words on paper, etc. was

1	2	3	4	5
No interference<br><br>(able see clearly past the static)	Weak interference	Mild interference<br><br>(most of the time able to see past the static but with mild difficulty)	Moderate interference	Severe interference<br><br>(never able to see clearly past the static)

CVSS (version 2.0, May 2023). All rights reserved. Email Victoria.Pelak@CUAnschutz.edu for permissions.

D. Reduction of daily activities: In the past month, the **average** degree the **visual static** reduced my ability to perform my daily activities was

1	2	3	4	5
No reduction<br><br>(the static never reduced my ability to perform activities at any time)	Minimal reduction<br><br>(the static reduced my ability to perform activities between 1% and 25% of the time)	Mild reduction<br><br>(the static reduced my ability to perform activities greater than 25% of the time but less than 50% of the time)	Moderate reduction<br><br>(the static reduced my ability to perform activities greater than 50% of the time but less than 75% of the time)	Severe reduction<br><br>(the static reduced my ability to perform activities greater than 75% of the time)

Section 2. Afterimages.

Definitions

- **Negative visual afterimage:** after shifting gaze away from viewing an object, there is a continued perception of the object appearing as the opposite color of the object.
- **Positive visual afterimage:** after shifting gaze away from viewing an object, there is a continued perception of the object appearing as the same color as the object.

I have experienced seeing positive and/or negative afterimages in the past month: Yes or No (if no, move to next section)

A. Intensity of Negative and/or **Positive Afterimages**: In the past month, the **average** visual intensity of the **afterimages** I experienced was

1	2	3	4	5
Not intense<br><br>(never vivid and lasted **less than** 60 seconds if negative **and less than** 1 second if positive)	Minimally intense<br><br>(vivid at times **and** lasted **less than** 60 seconds if negative and **less than** 1 second if positive)	Mildly intense<br><br>(vivid at times and lasted **greater than** 60 seconds if negative or **greater than** 1 second if positive)	Moderately intense<br><br>(vivid in most instances and lasted **greater than** 60 seconds if negative or **greater than** 1 second if positive)	Severely intense<br><br>(vivid in all instances and lasted **greater than** 60 seconds if negative or **greater than** 1 second if positive)

CVSS (version 2.0, May 2023). All rights reserved. Email Victoria.Pelak@CUAnschutz.edu for permissions.

B. Interference with vision while viewing electronic devices: In the past month, the **average** degree that the **afterimages** interfered with my vision after looking at electronic devices (computer or TV screen) was

1	2	3	4	5
No interference<br><br>(able see clearly during the afterimages without difficulty)	Minimal interference<br><br>(able to see during the afterimages with minimal difficulty)	Mild interference<br><br>(able to see during the afterimages with mild difficulty)	Moderate interference<br><br>(able to see during the afterimages with moderate difficulty)	Severe interference<br><br>(never able to see clearly during the afterimages)

C. Interference with vision while viewing the environment: In the past month, the **average** degree that the **afterimages** interfered with my ability to see things in my environment, **not** related to electronic devices, such as faces, objects, written words on paper, etc., was

1	2	3	4	5
No interference<br><br>(able see clearly during the afterimages without difficulty)	Weak interference	Mild interference<br><br>(able to see during the afterimages but with mild difficulty)	Moderate interference	Severe interference<br><br>(never able to see clearly during the afterimages)

D. Reduction of daily activities: In the past month, the **average** degree that the **afterimages** reduced my ability to perform my daily activities was

1	2	3	4	5
No reduction<br><br>(the afterimages never reduced my ability to perform activities at any time)	Minimal reduction<br><br>(the afterimages reduced my ability to perform activities between 1% and 25% of the time)	Mild reduction<br><br>(the afterimages reduced my ability to perform activities greater than 25% of the time but less than 50% of the time)	Moderate reduction<br><br>(the afterimages reduced my ability to perform activities greater than 50% of the time but less than 75% of the time)	Severe reduction<br><br>(the afterimages reduced my ability to perform activities greater than 75% of the time)

CVSS (version 2.0, May 2023). All rights reserved. Email Victoria.Pelak@CUAnschutz.edu for permissions.

Section 3. Trails.

Definitions:

- **Visual Trails:** While viewing an object in motion, there is a series of images of the object that follow the object as it moves and then the images fade out along the object's path.

I have experienced seeing visual trails in the last month: Yes or No (if no, move to next section)

A. Intensity of Trails: In the past month, the **average** visual intensity of the **trails** that I experienced was

1	2	3	4	5
Not intense<br><br>(the trails were never vivid **and/or** always less than 10 seconds)	Minimally intense<br><br>(in a few instances, the trails were vivid **and** greater than 10 seconds)	Mildly intense<br><br>(as often as not, the trails were vivid **and** greater than 10 seconds)	Moderately intense<br><br>(in most instances, the trails were vivid **and** greater than 10 seconds)	Severely intense<br><br>(in all instances, the trails were vivid **and** greater than 10 seconds)

B. Interference with vision while viewing electronic devices: In the past month, the **average** degree that the **trails** interfered with my vision while using electronic devices (computer or TV screen) was

1	2	3	4	5
No interference<br><br>(able see clearly with trails without difficulty)	Minimal interference<br><br>(able to see during the trails and with minimal difficulty)	Mild interference<br><br>(able to see during the trails but with mild difficulty)	Moderate interference<br><br>(able to see during the trails but with moderate difficulty)	Severe interference<br><br>(never able to see clearly during the trails)

CVSS (version 2.0, May 2023). All rights reserved. Email Victoria.Pelak@CUAnschutz.edu for permissions.

C. Interference with vision while viewing the environment: In the past month, the **average** degree that the **trails** interfered with my ability to see things in my environment, **not** related to electronic devices, such as faces, objects, written words on paper, etc., was

1	2	3	4	5
No interference<br><br>(able see clearly regardless of trails)	Weak interference	Mild interference<br><br>(able to see during the trails but with mild difficulty)	Moderate interference	Severe interference<br><br>(never able to see clearly during the trails)

D. Reduction of daily activities: In the past month, the **average** degree that the **trails** reduced my ability to perform my daily activities was

1	2	3	4	5
No reduction<br><br>(the trails never reduced my ability to perform activities at any time)	Minimal reduction<br><br>(the trails reduced my ability to perform activities between 1% and 25% of the time)	Mild reduction<br><br>(the trails reduced my ability to perform activities greater than 25% of the time but less than 50% of the time)	Moderate reduction<br><br>(the trails reduced my ability to perform activities greater than 50% of the time but less than 75% of the time)	Severe reduction<br><br>(the trails reduced my ability to perform activities greater than 75% of the time)

CVSS (version 2.0, May 2023). All rights reserved. Email Victoria.Pelak@CUAnschutz.edu for permissions.

Section 4. Blue field entoptic phenomenon.

Definitions:

- **Blue field entoptic phenomenon**: While looking at a bright blue background like the sky, small bright dots appear and move quickly along linear or snaking paths in the visual field.

I have experienced blue field entoptic phenomenon in the past month: Yes or No (if no, move to next section)

A. Visual Intensity: In the past month, the **average** visual intensity of the **blue field entoptic phenomenon** that I experienced was

1	2	3	4	5
Not intense<br><br>(the phenomenon was not distinct and was muted or dim)	Weakly intense	Mildly intense<br><br>(the phenomenon was distinct but not vivid or bright)	Moderately intense	Severely intense<br><br>(the phenomenon was distinct and is vivid or bright)

B. Interference with vision while viewing electronic devices: In the past month, the **average** degree that **blue field entoptic phenomenon** interfered with my vision while looking at electronic devices (smart phone, tablet, computer, TV screen, etc.) was

1	2	3	4	5
No interference<br><br>(able see clearly past the phenomenon)	Weak interference	Mild interference<br><br>(able to see past the phenomenon but with mild difficulty)	Moderate interference	Severe interference<br><br>(never able to see clearly past the phenomenon)

C. Interference with vision while viewing the environment: In the past month, the **average** degree that the **blue field entoptic phenomenon** interfered with my ability to see things in my environment, **not** related to electronic devices, such as faces, objects, written words on paper, etc., was

1	2	3	4	5
No interference<br><br>(able see clearly past the phenomenon with no difficulty)	Weak interference	Mild interference<br><br>(able to see past the phenomenon but with mild difficulty)	Moderate interference	Severe interference<br><br>(never able to see clearly past the phenomenon)

CVSS (version 2.0, May 2023). All rights reserved. Email Victoria.Pelak@CUAnschutz.edu for permissions.

D. Reduction of daily activities: In the past month, the **average** degree that the **blue field entoptic phenomenon** reduced my ability to perform my daily activities was

1	2	3	4	5
No reduction<br><br>(the phenomenon never reduced my ability to perform activities at any time)	Minimal reduction<br><br>(the phenomenon reduced my ability to perform activities between 1% and 25% of the time)	Mild reduction<br><br>(the phenomenon reduced my ability to perform activities greater than 25% of the time but less than 50% of the time)	Moderate reduction<br><br>(the phenomenon reduced my ability to perform activities greater than 50% of the time but less than 75% of the time)	Severe reduction<br><br>(the phenomenon reduced my ability to perform activities greater than 75% of the time)

Section 5. Floaters.

Definitions:

- **Floaters**: small, dark or semi-transparent specks, spots, or dots; floaters can also appear as thread-like or cobweb-like shapes; floaters are seen from within the eye and float or drift with your eye movements.

I have experienced seeing floaters in the past month: Yes or No (if no, move to next section)

A. Visual Intensity: In the past month, the **average** visual intensity of the **floaters** that I experienced was

1	2	3	4	5
Not intense<br><br>(the floaters were not distinct, not numerous, and easy to ignore)	Weakly intense	Mildly intense<br><br>(most of the time the floaters were distinct, but not numerous, and could be ignored)	Moderately intense	Severely intense<br><br>(the floaters were distinct, very numerous, and could not be ignored)

CVSS (version 2.0, May 2023). All rights reserved. Email Victoria.Pelak@CUAnschutz.edu for permissions.

B. Interference with vision while viewing electronic devices: In the past month, the **average** degree that **floaters** interfered with my vision while looking at electronic devices (smart phone, tablet, computer, TV screen, etc.) was

1	2	3	4	5
No interference<br><br>(able see clearly past the floaters)	Weak interference	Mild interference<br><br>(able to see past the floaters but with mild difficulty)	Moderate interference	Severe interference<br><br>(never able to see clearly past the floaters)

C. Interference with vision while viewing the environment In the past month, the **average** degree that the **floaters** interfered with my ability to see things in my environment, **not** related to electronic devices, such as faces, objects, written words on paper, etc., was

1	2	3	4	5
No interference<br><br>(able see clearly past the floaters)	Weak interference	Mild interference<br><br>(able to see past the floaters but with mild difficulty)	Moderate interference	Severe interference<br><br>(never able to see clearly past the floaters)

D. Reduction of daily activities: In the past month, the **average** degree that the **floaters** reduced my ability to perform my daily activities was

1	2	3	4	5
No reduction<br><br>(the floaters never reduced my ability to perform activities at any time)	Minimal reduction<br><br>(the floaters reduced my ability to perform activities between 1% and 25% of the time)	Mild reduction<br><br>(the floaters reduced my ability to perform activities greater than 25% of the time but less than 50% of the time)	Moderate reduction<br><br>(the floaters reduced my ability to perform activities greater than 50% of the time but less than 75% of the time)	Severe reduction<br><br>(the floaters reduced my ability to perform activities greater than 75% of the time)

CVSS (version 2.0, May 2023). All rights reserved. Email Victoria.Pelak@CUAnschutz.edu for permissions.

Section 6. Night vision problems.

Definition:

- **Night vision problems:** Problems with vision at night that started **WITH** or **AFTER** the visual snow or static symptoms started.

I have experienced night vision problems in the past month: Yes or No (if no, move to next section)

A. Overall Intensity: In the past month, the **average** intensity of the **night vision problems** that I have experienced was

1	2	3	4	5
Not intense<br><br>(not noticeable **and/or** can adjust to dark immediately)	Weakly intense	Mildly intense<br><br>(most of the time I was able to ignore the night vision problems **and/or** adjust to dark with mild difficulty)	Moderately intense	Severely intense<br><br>(never able to ignore the night vision problems **and/or** adjust to the dark)

B. Interference with vision while viewing electronic devices: In the past month, the **average** degree that **night vision problems** interfered with my vision while looking at electronic devices (smart phone, tablet, computer, TV screen, etc.) was

1	2	3	4	5
No interference<br><br>(able see clearly at night on a device)	Weak interference	Mild interference<br><br>(most of the time I was able to see clearly at night on a device but with mild difficulty)	Moderate interference	Severe interference<br><br>(never able to see clearly at night on a device)

CVSS (version 2.0, May 2023). All rights reserved. Email Victoria.Pelak@CUAnschutz.edu for permissions.

C. Interference with vision while viewing the environment: In the past month, the **average** degree that the **night vision problems** interfered with my ability to see things in my environment, not related to electronic devices, such as faces, objects, written words on paper, etc., was

1	2	3	4	5
No interference<br><br>(able see clearly at night)	Weak interference	Mild interference<br><br>(most of the time I was able to see at night but with mild difficulty)	Moderate interference	Severe interference<br><br>(never able to see clearly at night)

D. Reduction of daily (nighttime) activities: In the past month, the **average** degree that the **night vision problems** reduced my ability to perform activities at night was

1	2	3	4	5
No reduction<br><br>(the night vision issues never reduced my ability to perform activities at any time)	Minimal reduction<br><br>(the night vision issues reduced my ability to perform activities between 1% and 25% of the time)	Mild reduction<br><br>(the night vision issues reduced my ability to perform activities greater than 25% of the time but less than 50% of the time)	Moderate reduction<br><br>(the night vision issues reduced my ability to perform activities greater than 50% of the time but less than 75% of the time)	Severe reduction<br><br>(the night vision issues reduced my ability to perform activities greater than 75% of the time)

CVSS (version 2.0, May 2023). All rights reserved. Email Victoria.Pelak@CUAnschutz.edu for permissions.

Section 7. Ringing in the ears or Tinnitus.

Definition:

- **Tinnitus:** The sound of ringing or buzzing in the head and/or in one or both ears without an external source.

I have experienced tinnitus in the past month: Yes or No (if no, move to next section)

A. Overall Intensity: In the past month, the **average** intensity of the **tinnitus** that I experienced was

1	2	3	4	5
Not intense<br><br>(not noticeable **and/or** I adjusted immediately)	Weakly intense	Mildly intense<br><br>(most of the time I was able to ignore **and/or** adjust with only mild difficulty)	Moderately intense	Severely intense<br><br>(never able to ignore **and/or** adjust)

B. Interference with hearing: In the past month, the **average** degree that the **tinnitus** interfered with my ability to hear was

1	2	3	4	5
No interference<br><br>(not noticeable **and/or** I adjusted immediately)	Weak interference	Mild interference<br><br>(most of the time I was able to ignore **and/or** adjust with only mild difficulty)	Moderate interference	Severe interference<br><br>(never able to ignore **and/or** adjust)

C. Interference with sleep: In the past month, the **average** degree that the **tinnitus** interfered with my sleep was

1	2	3	4	5
No interference<br><br>(never interfered my sleep)	Minimal interference<br><br>(interfered with my sleep up to 25% of the time)	Mild interference<br><br>(interfered with my sleep greater than 25% and less than 50% of the time and/or degree)	Moderate interference<br><br>(interfered my sleep between 50-75% of the time)	Severe interference<br><br>(interfered with my sleep greater than 75% of the time)

CVSS (version 2.0, May 2023). All rights reserved. Email Victoria.Pelak@CUAnschutz.edu for permissions.

D. Reduction of daily activities: In the past month, the **average** degree that the **tinnitus** reduced my ability to perform my daily activities was

1	2	3	4	5
No reduction<br><br>(never reduced my ability to perform activities at any time)	Minimal reduction<br><br>(reduced my ability to perform activities between 1% and 25% of the time)	Mild reduction<br><br>(reduced my ability to perform activities greater than 25% of the time but less than 50% of the time)	Moderate reduction<br><br>(reduced my ability to perform activities greater than 50% of the time but less than 75% of the time)	Severe reduction<br><br>(reduced my ability to perform activities greater than 75% of the time)

Section 8. Depersonalization and derealization.

Definition:

- **Depersonalization**: The feeling of being detached or separate from your thoughts, feelings, and body.
- **Derealization**: The feeling of being disconnected from your environment.
- **Wellness**: The feeling or quality of being in a good state of health.

I have experienced depersonalization or derealization in the past month: Yes or No (if no, move to next section)

A. Overall Intensity: In the past month, the **average** intensity of the **feeling of depersonalization or derealization** that I experienced was

1	2	3	4	5
Not intense<br><br>(not noticeable **and/or** can adjust immediately)	Weakly intense	Mildly intense<br><br>(most of the time I was able to ignore **and/or** adjust with mild difficulty)	Moderately intense	Severely intense<br><br>(never able to ignore **and/or** adjust)

CVSS (version 2.0, May 2023). All rights reserved. Email Victoria.Pelak@CUAnschutz.edu for permissions.

B. Interference with socializing: In the past month, the **average** degree that the **feelings of depersonalization or derealization** interfered with my ability to socialize was

1	2	3	4	5
No interference<br><br>(not noticeable **and/or** I adjusted immediately)	Weak interference	Mild interference<br><br>(most of the time I was able to ignore **and/or** adjust with only mild difficulty)	Moderate interference	Severe interference<br><br>(never able to ignore **and/or** adjust)

C. Interference with feelings of wellness. In the past month, the **average** degree that the feelings of **depersonalization or derealization** interfered with my feeling of wellness was

1	2	3	4	5
No interference<br><br>(not noticeable **and/or** did not impact my wellness)	Minimal interference<br><br>(interfered with my wellness up to 25% of the time)	Mild interference<br><br>(interfered with my wellness greater than 25% and less than 50% of the time)	Moderate interference<br><br>(interfered with my wellness between 50-75% of the time)	Severe interference<br><br>(interfered with my wellness greater than 75% of the time)

D. Reduction of daily activities: In the past month, the **average** degree the **depersonalization or derealization** reduced my ability to perform my daily activities was

1	2	3	4	5
No reduction<br><br>(never reduced my ability to perform activities at any time)	Minimal reduction<br><br>(reduced my ability to perform activities between 1% and 25% of the time	Mild reduction<br><br>(reduced my ability to perform activities greater than 25% of the time but less than 50% of the time)	Moderate reduction<br><br>(reduced my ability to perform activities greater than 50% of the time but less than 75% of the time)	Severe reduction<br><br>(reduced my ability to perform activities greater than 75% of the time)

CVSS (version 2.0, May 2023). All rights reserved. Email Victoria.Pelak@CUAnschutz.edu for permissions.

Section 9. Anxiety.

Definition:

- **Anxiety**: Anxiety is uncontrollable feelings of anxiety, fear, worry, and/or panic.
- **Wellness**: The feeling or quality of being in a good state of health.

I have experienced anxiety in the past month: Yes or No (if no, move to next section)

A. Overall Intensity: In the past month, the **average** intensity of **anxiety** that I experienced was

1	2	3	4	5
Not intense<br><br>(not noticeable **and/or** can adjust immediately)	Weakly intense	Mildly intense<br><br>(most of the time I was able to ignore **and/or** adjust with mild difficulty)	Moderately intense	Severely intense<br><br>(never able to ignore **and/or** adjust)

B. Interference with socializing: In the past month, the **average** degree that **anxiety** interfered with my ability to socialize was

1	2	3	4	5
No interference<br><br>(not noticeable **and/or** I adjusted immediately)	Weak interference	Mild interference<br><br>(most of the time I was able to ignore **and/or** adjust with only mild difficulty)	Moderate interference	Severe interference<br><br>(never able to ignore **and/or** adjust)

C. Interference with feelings of wellness. In the past month, the **average** degree that **anxiety** interfered with my feeling of wellness:

1	2	3	4	5
No interference<br><br>(not noticeable **and/or** did not impact my wellness)	Minimal interference<br><br>(interfered with my wellness up to 25% of the time)	Mild interference<br><br>(interfered with my wellness greater than 25% and less than 50% of the time)	Moderate interference<br><br>(interfered with my wellness between 50-75% of the time)	Severe interference<br><br>(interfered with my wellness greater than 75% of the time)

CVSS (version 2.0, May 2023). All rights reserved. Email Victoria.Pelak@CUAnschutz.edu for permissions.

D. Reduction of daily activities: In the past month, the **average** degree that **anxiety** reduced my ability to perform my daily activities was

1	2	3	4	5
No reduction<br><br>(never reduced my ability to perform activities at any time)	Minimal reduction<br><br>(reduced my ability to perform activities between 1% and 25% of the time)	Mild reduction<br><br>(reduced my ability to perform activities greater than 25% of the time but less than 50% of the time)	Moderate reduction<br><br>(reduced my ability to perform activities greater than 50% of the time but less than 75% of the time)	Severe reduction<br><br>(reduced my ability to perform activities greater than 75% of the time)

Section 10. Symptoms of sadness or loss of interest.

Definitions:

- **Sadness**: A feeling of being unhappy.
- **Loss of interest:** No longer interested in activities that you usually find enjoyable.

I have experienced symptoms of sadness or loss of interest in the past month: Yes or No (if no, move to next section)

A. Overall Intensity: In the past month, the **average** intensity **of sadness or loss of interest** that I experienced was

1	2	3	4	5
Not intense<br><br>(not noticeable **and/or** can adjust immediately)	Weakly intense	Mildly intense<br><br>(most of the time I was able to ignore **and/or** adjust with mild difficulty)	Moderately intense	Severely intense<br><br>(never able to ignore **and/or** adjust)

CVSS (version 2.0, May 2023). All rights reserved. Email Victoria.Pelak@CUAnschutz.edu for permissions.

D. I**nterference with socializing**: In the past month, the **average** degree that **sadness or loss of interest** interfered with my ability to socialize was

1	2	3	4	5
No interference<br><br>(not noticeable **and/or** I adjusted immediately)	Weak interference	Mild interference<br><br>(most of the time I was able to ignore **and/or** adjust with only mild difficulty)	Moderate interference	Severe interference<br><br>(never able to ignore **and/or** adjust)

C. Interference with feelings of wellness. In the past month, the **average** degree **that sadness or loss of interest** interfered with my feeling of wellness was

1	2	3	4	5
No interference<br><br>(not noticeable **and/or** did not impact my wellness)	Minimal interference<br><br>(interfered with my wellness up to 25% of the time)	Mild interference<br><br>(interfered with my wellness greater than 25% and less than 50% of the time)	Moderate interference<br><br>(interfered with my wellness between 50-75% of the time)	Severe interference<br><br>(interfered with my wellness greater than 75% of the time)

D. Reduction of daily activities: In the past month, the **average** degree that that **sadness or loss of interest** reduced my ability to perform my daily activities was

1	2	3	4	5
No reduction<br><br>(never reduced my ability to perform activities at any time)	Minimal reduction<br><br>(reduced my ability to perform activities between 1% and 25% of the time)	Mild reduction<br><br>(reduced my ability to perform activities greater than 25% of the time but less than 50% of the time)	Moderate reduction<br><br>(reduced my ability to perform activities greater than 50% of the time but less than 75% of the time)	Severe reduction<br><br>(reduced my ability to perform activities greater than 75% of the time)

CVSS (version 2.0, May 2023). All rights reserved. Email Victoria.Pelak@CUAnschutz.edu for permissions.

CVSS PART 2.

CVSS Sense of Change over the past month

Before filling out the table, please wait for instructions.

**Symptoms**	**Symptom worsened or newly developed or returned during the past month after being absent** (-1 point will be given for each answer in this column)	**Symptom did not change during the past month** (0 points will be given for each answer in this column)	**Symptom improved or stopped during the past month after being present** (+1 point for each answer in this column)	**Never had this symptom during the past month** (0 points for each answer in this column)
Visual static				Not a choice
Afterimages				
Trails				
Blue field entoptic phenomenon				
Floaters				
Night vision problems				
Tinnitus				
Depersonalization or derealization				
Anxiety				
Sadness or loss of interest				
