# Supplementary material for: Assessment of a novel patient reported outcome measure for visual snow syndrome: the Colorado visual snow survey 2.0
Source: Front Neurol. 2025 Sep 29;16:1664310. doi: 10.3389/fneur.2025.1664310 (PMC12515653; doi:10.3389/fneur.2025.1664310)
Supplement: Supplementary file 2 [file Table_1.docx]

**Table S1.** *Average score by CVSS metric and group.* Significance first tested for a group effect, significance listed under ‘Metric’ column. Pairwise significance also assessed. ‘VSS D’ refers to the VSS self-reported diagnosed group, ‘VSS UD’ refers to the self-reported undiagnosed group. Intensity refers to subquestion A, digital interference refers to subquestion B, environment interference refers to subquestion C, and daily activities reduction refers to subquestion D. Wording is different for the last four metrics, see CVSS for details. P values displayed after Greenhouse-Geisser correction. After Bonferroni correction, significance threshold is p < 0.00125. NS: p > .0025; + < .0025; * < .00125; ** < .00025; *** < .000025.

| Metric (Group effect) | VSS (Total) (D-UD Comparison) | VSS-D (D-Control Comparison) | VSS-UD (UD-Control Comparison) | Control |
| --- | --- | --- | --- | --- |
| Visual static |  |  |  |  |
| Intensity (***) | 3.29 (NS) | 3.18 (***) | 3.41 (***) | 0.05 |
| Digital interference (***) | 2.98 (NS) | 2.94 (***) | 3.03 (***) | 0.04 |
| Environment interference (***) | 2.92 (NS) | 2.82 (***) | 3.03 (***) | 0.05 |
| Daily activities reduction (***) | 2.35 (NS) | 2.53 (***) | 2.16 (***) | 0.06 |
| Afterimages |  |  |  |  |
| Intensity (***) | 2.17 (NS) | 2.38 (***) | 1.94 (***) | 0.35 |
| Digital interference (***) | 2.32 (NS) | 2.65 (***) | 1.97 (***) | 0.40 |
| Environment interference (***) | 2.41 (NS) | 2.76 (***) | 2.03 (***) | 0.35 |
| Daily activities reduction (***) | 1.95 (NS) | 2.18 (***) | 1.72 (***) | 0.26 |
| Trails |  |  |  |  |
| Intensity (***) | 1.17 (NS) | 1.38 (***) | 0.94 (*) | 0.10 |
| Digital interference (***) | 1.21 (NS) | 1.44 (***) | 0.97 (*) | 0.12 |
| Environment interference (***) | 1.41 (NS) | 1.62 (***) | 1.19 (***) | 0.09 |
| Daily activities reduction (***) | 1.2 (NS) | 1.44 (***) | 0.94 (**) | 0.08 |
| Blue field entoptic phenomenon |  |  |  |  |
| Intensity (***) | 2.71 (NS) | 2.74 (***) | 2.69 (***) | 0.41 |
| Digital interference (***) | 1.85 (NS) | 1.94 (***) | 1.75 (***) | 0.29 |
| Environment interference (***) | 2.15 (NS) | 2.24 (***) | 2.06 (***) | 0.27 |
| Daily activities reduction (***) | 1.5 (NS) | 1.62 (***) | 1.38 (***) | 0.22 |
| Floaters |  |  |  |  |
| Intensity (***) | 2.58 (NS) | 2.32 (**) | 2.84 (***) | 0.92 |
| Digital interference (***) | 2.03 (NS) | 1.88 (**) | 2.19 (***) | 0.78 |
| Environment interference (***) | 2.12 (NS) | 2 (***) | 2.25 (***) | 0.73 |
| Daily activities reduction (***) | 1.62 (NS) | 1.47 (**) | 1.78 (***) | 0.55 |
| Diminished night vision |  |  |  |  |
| Intensity (***) | 3.21 (NS) | 3.12 (***) | 3.31 (***) | 0.33 |
| Digital interference (***) | 2.3 (NS) | 2.35 (***) | 2.25 (***) | 0.31 |
| Environment interference (***) | 3.12 (NS) | 3.06 (***) | 3.19 (***) | 0.41 |
| Daily activities reduction (***) | 2.64 (NS) | 2.62 (***) | 2.66 (***) | 0.24 |
| Tinnitus |  |  |  |  |
| Intensity (***) | 2.47 (NS) | 2.29 (NS) | 2.66 (**) | 1.31 |
| Hearing interference (***) | 2.05 (NS) | 1.88 (+) | 2.22 (***) | 0.97 |
| Sleeping interference (***) | 1.68 (NS) | 1.76 (***) | 1.59 (***) | 0.63 |
| Daily activities reduction (***) | 1.38 (NS) | 1.12 (NS) | 1.66 (***) | 0.73 |
| Depersonalization/ derealization |  |  |  |  |
| Intensity (***) | 2.23 (NS) | 1.91 (NS) | 2.56 (***) | 0.90 |
| Socializing interference (***) | 2.24 (NS) | 2.00 (*) | 2.5 (***) | 0.85 |
| Wellness interference (***) | 2.18 (NS) | 2.00 (*) | 2.38 (***) | 0.77 |
| Daily activities reduction (***) | 1.95 (NS) | 1.68 (*) | 2.25 (***) | 0.64 |
| Anxiety |  |  |  |  |
| Intensity (NS) | 3.24 (NS) | 3.29 (NS) | 3.19 (NS) | 2.63 |
| Socializing interference (NS) | 2.94 (NS) | 2.82 (NS) | 3.06 (NS) | 2.42 |
| Wellness interference (*) | 3.11 (NS) | 3.09 (NS) | 3.12 (NS) | 2.23 |
| Daily activities reduction (NS) | 2.65 (NS) | 2.47 (NS) | 2.84 (NS) | 2.06 |
| Sadness/loss of interest |  |  |  |  |
| Intensity (NS) | 2.71 (NS) | 2.62 (NS) | 2.81 (NS) | 1.74 |
| Socializing interference (*) | 2.56 (NS) | 2.35 (NS) | 2.78 (*) | 1.49 |
| Wellness interference (**) | 2.71 (NS) | 2.59 (NS) | 2.84 (*) | 1.49 |
| Daily activities reduction (**) | 2.38 (NS) | 2.18 (NS) | 2.59 (**) | 1.31 |

**Table S2.** *Loadings of each CVSS symptom (when visual static omitted from data).* Factor 1 explained 42.8% of variance. Factor 2 explained 11.8% of variance.

|  | Factor 1 | Factor 2 |
| --- | --- | --- |
| Afterimages | 0.20 | -0.13 |
| Trails | 0.17 | -0.06 |
| Blue field entoptic phenomenon | 0.19 | -0.06 |
| Floaters | 0.16 | -0.12 |
| Diminished night vision | 0.18 | -0.16 |
| Tinnitus | 0.14 | -0.09 |
| Depersonalization/ derealization | 0.16 | 0.19 |
| Anxiety | 0.13 | 0.27 |
| Sadness/loss of interest | 0.16 | 0.27 |

**Table S3.** *Zero-order Spearman correlations with age on symptom intensity in full VSS sample (N = 64; two subjects omitted age).*

|  | Spearman Rho [95% CI] | *P* Value |
| --- | --- | --- |
| Visual static | 0.09 [-0.16, 0.34] | 0.45 |
| Afterimages | 0.35 [0.11, 0.55] | **<0.01** |
| Trails | -0.17 [-0.40, 0.08] | 0.19 |
| Blue field entoptic phenomenon | 0.06 [-0.19, 0.31] | 0.63 |
| Floaters | 0.24 [-0.01, 0.46] | 0.06 |
| Diminished night vision | 0.24 [-0.01, 0.46] | 0.06 |
| Tinnitus | 0.20 [-0.05, 0.43] | 0.12 |
| Depersonalization/ derealization | 0.21 [-0.05, 0.43] | 0.11 |
| Anxiety | 0.07 [-0.18, 0.32] | 0.58 |
| Sadness/loss of interest | 0.26 [0.01, 0.48] | **0.03** |
